# Molecular Rotors Detect the Formation and Conversion of α-Synuclein Oligomers

**DOI:** 10.1021/acsami.4c21710

**Published:** 2025-02-05

**Authors:** Siân C. Allerton, Marina K. Kuimova, Francesco A. Aprile

**Affiliations:** † Department of Chemistry, Molecular Sciences Research Hub, Imperial College London, London W12 0BZ, U.K.; ‡ Institute of Chemical Biology, Molecular Sciences Research Hub, Imperial College London, London W12 0BZ, U.K.

**Keywords:** α-synuclein, amyloids, oligomers, molecular rotors, fluorescence lifetime

## Abstract

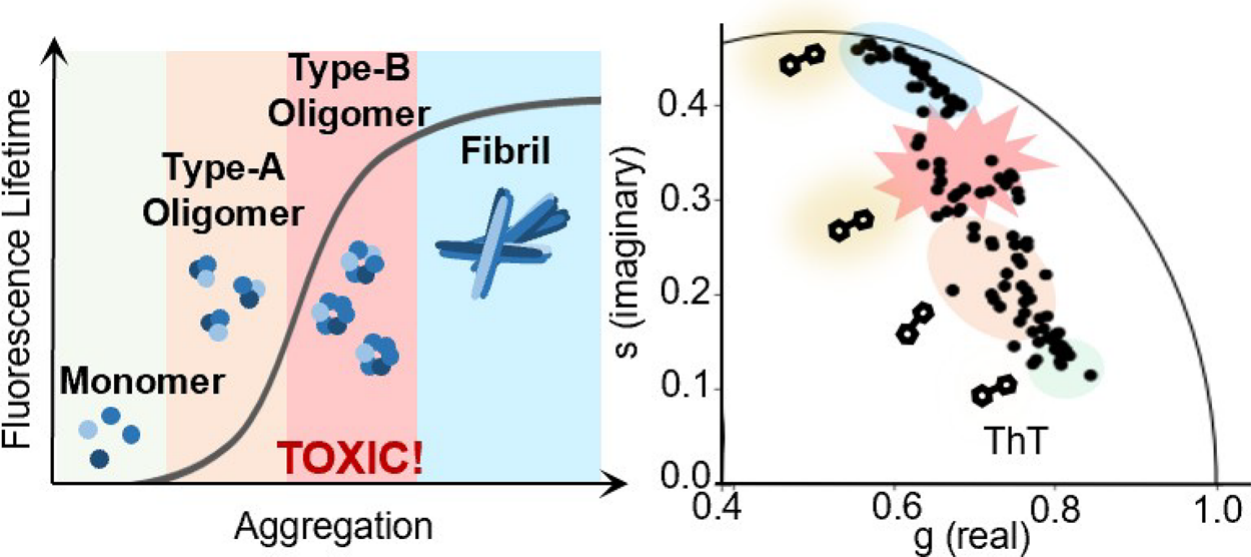

α-Synuclein is an intrinsically disordered protein that forms amyloids in Parkinson’s disease. Currently, detection methods predominantly report on the formation of mature amyloids but are weakly sensitive to the early stage, toxic oligomers. Molecular rotors are fluorophores that sense changes in the viscosity of their local environment. Here, we monitor α-synuclein oligomer formation using the fluorescence lifetime of molecular rotors. We detect oligomer formation and conversion into amyloids for wild-type and two α-synuclein variants, the pathological mutant A30P and ΔP1 α-synuclein, which lacks a master regulator region of aggregation (residues 36–42). We report that A30P α-synuclein shows a rate of oligomer formation similar to that of wild-type α-synuclein, whereas ΔP1 α-synuclein shows delayed oligomer formation. Additionally, both variants demonstrate a slower conversion of oligomers into amyloids. Our method provides a quantitative approach to unveiling the complex mechanism of α-synuclein aggregation, which is key to understanding the pathology of Parkinson’s disease.

## Introduction

Second only to Alzheimer’s disease (AD), Parkinson’s disease (PD) is the most prevalent neurodegenerative age-associated disorder.^1−3^ PD is characterized by the formation of intraneuronal inclusions, the Lewy bodies (LB), which are predominantly composed of aggregates of the intrinsically disordered protein α-synuclein (αSyn).^1,4−9^ The majority of these aggregates, called amyloids, have a fibrillar shape and are enriched β-sheets.^2,3,10^ Although αSyn plays a role in the pathology of PD, this 14.5 kDa protein, located at the presynaptic terminals of neurons,^11,12^ is key to multiple physiological processes including neurotransmitter synthesis and release, and synaptic vesicle recycling.^13−16^

Recent studies indicate that oligomers, which form earlier in the aggregation process, are more toxic than amyloid fibrils and may be responsible for the disease onset and progression.^3,5,17^ These oligomers are highly heterogeneous in terms of conformation and toxic mechanisms.

Currently, the standard method for monitoring the aggregation of αSyn is by fluorescence intensity assays using switch-on dyes such as Thioflavin T (ThT) and PROTEOSTAT.^18−20^ However, this method predominantly reports on the formation of mature amyloid fibrils and is poorly sensitive to oligomers, unless super resolution techniques are employed.^17,18,21^ Fluorescence lifetime imaging microscopy (FLIM) can monitor the distribution of decay time in a spatial and time-resolved manner, therefore enabling the detection of photophysical events which fluorescence intensity imaging cannot achieve.

We set out to investigate whether oligomer detection can be achieved using molecular rotors (MRs).^18,22^ MRs display high nonradiative decay rates upon excitation in low-viscosity environments due to unrestricted rotation about an intramolecular bond. In contrast, in more viscous or crowded environments, the rotation is restricted, causing a decrease in the nonradiative decay rate. This results in high fluorescence intensity and long fluorescence lifetimes, the latter is particularly suited to concentration-independent monitoring.^18,22,23^ In a crowded protein solution, the fluorescence lifetime of a MR can change to reflect the microviscosity of the environment, or due to crowding of a MR in the presence of different structural species formed during the aggregation.

We previously demonstrated that the MRs, ThT and 3,3′- diethylthiacarbocyanine iodide (DiSC_2_) were able to follow the aggregation pathway of lysozyme and insulin, since aggregation caused the solution free volume sensed by these rotors to reduce, resulting in longer fluorescence lifetimes. Interestingly, the aggregation trajectory sensed by the lifetimes of ThT and DiSC_2_ were complex, indicating that the rotor-based detection was sensitive to species other than just the monomers and fully formed fibrils.^18^

Here, we explore the exciting potential of MR intensity and lifetime-based techniques to reveal the complexities of the aggregation mechanisms for αSyn involving oligomeric species. This is particularly important as establishing methods to identify and monitor the formation of oligomers is key to developing therapeutic approaches to target these potentially toxic aggregates, aiding in the development of clinical strategies for PD.

## Results and Discussion

### The Fluorescence Lifetime of DiSC_2_ and ThT Increases before Intensity during WT αSyn Aggregation

**Figure 1:**
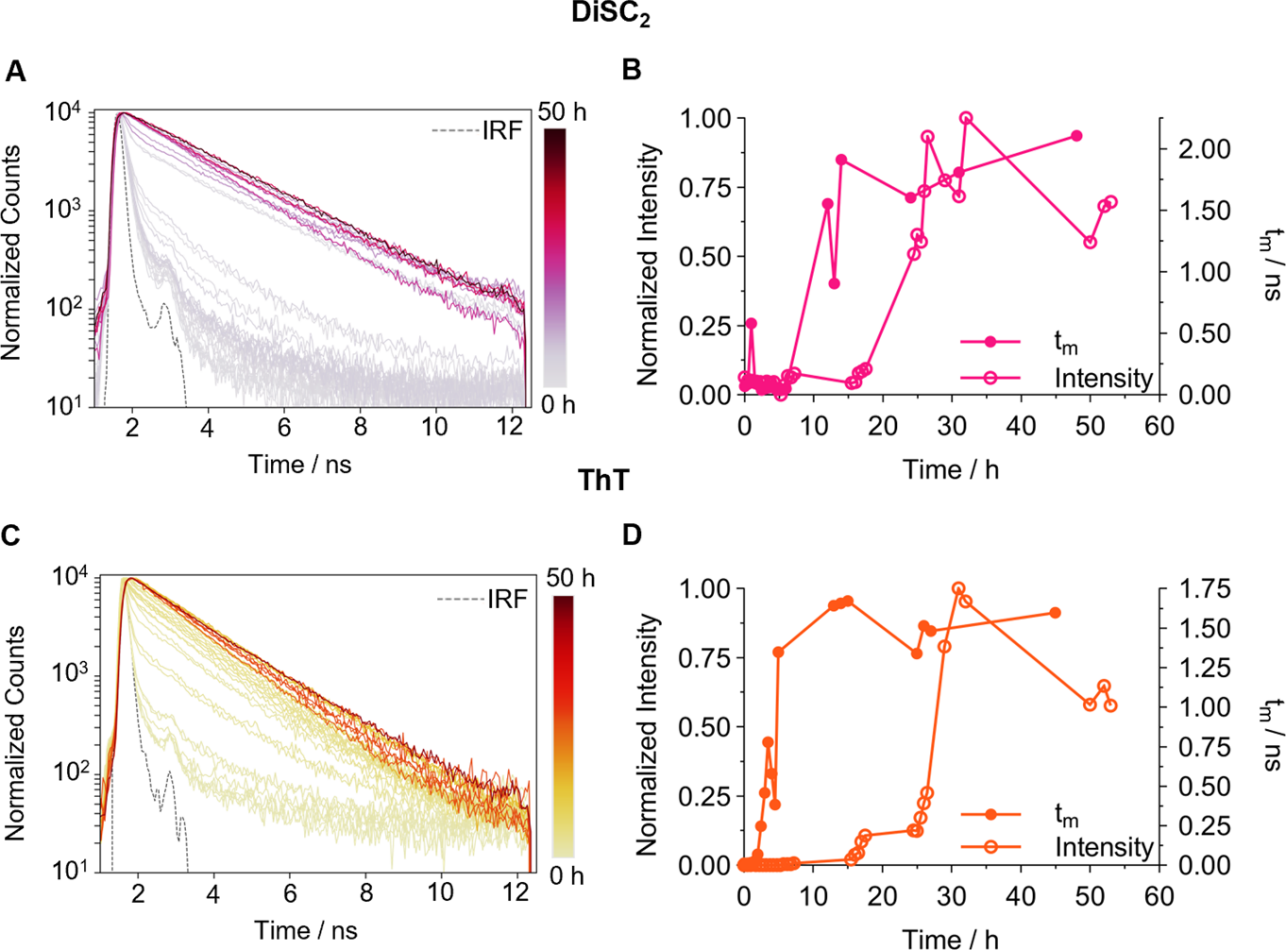
Time-resolved fluorescence decays and τm analysis of DiSC_2_ and ThT monitored during WT αSyn aggregation in PBS. (A) Single representative set of time-resolved fluorescence decays of DiSC_2_ (3 μM) recorded during WT αSyn (150 μM) aggregation. (B) Comparative studies of the intensity (empty circles) and τ_m_ (filled circles) calculated from the DiSC_2_ time-resolved decays in panel A. (C) Single representative set of time-resolved fluorescence decays of ThT (10 μM) recorded during WT αSyn (150 μM) aggregation. (D) Comparative studies of the intensity (empty circles) and τ_m_ (filled circles) calculated from the ThT time-resolved decays in panel C. The full data sets from all repeats are shown in Figures S4 (DiSC_2_) and S5 (ThT).

The time-resolved fluorescence decays of DiSC_2_ and ThT were measured during the aggregation of *wild-type* (WT) αSyn, alongside traditional intensity measurements (Figure 1 and Figures S1, S2, S4, and S5). We selected ThT and DiSC_2_ as our MRs as both dyes are known to interact with amyloid fibrils.^18,24,25^ The aggregations were carried out in a glass-bottomed 96-well plate which was agitated (450 rpm) in a benchtop incubator at 37 °C. At specific incubation times, the intensity (using a plate reader) and the time-resolved fluorescence decays (using a FLIM microscope) were recorded. Importantly, both DiSC_2_ and ThT show little change in their fluorescence lifetime in the aggregation conditions, when protein is not present (Figures S16–S18).

The fluorescence decays of DiSC_2_ and ThT (Figure 1A,C) are initially dominated by a short-time component. This is indicative of the MR being in a nonviscous environment, most likely free in solution with purely monomeric WT αSyn, which does not restrict the MRs rotation. As the aggregation proceeds, the fluorescence lifetime of both rotors increases. This was expected as the aggregating species cause higher crowding which results in increased confinement of the MRs. Alternatively, the MR may bind to certain type(s) of aggregates, restricting its nonradiative relaxation to ground state in the bound conformation. However, if this was the case, we would expect to detect biexponential decays with fixed components and varying amplitudes, due to unbound (short component) and bound (long component) conformers of the MR. This is not the case and therefore, we can exclude a simple binding model where MRs bind to a single species present in the aggregation mixture, *i.e*. WT αSyn mature fibrils.

Both MRs exhibit a similar rise in fluorescence intensity after ∼15 h, indicating the intensity of both is sensitive to the presence of mature fibrils, consistent with literature data for ThT.^19,26^ Interestingly, the amplitude-weighted average lifetime (τ_m_) of DiSC_2_ and ThT, plotted against aggregation time reveals a much earlier increase compared to fluorescence intensity (Figure 1B,D).

An increase in ThT τ_m_ is observed after 2 h of aggregation whereas the DiSC_2_ τ_m_ increases only after 12 h (Figure 1B,D). This data suggests that while the lifetime for both MRs can detect earlier formed species relative to intensity, ThT is more sensitive to these species compared to DiSC_2_. Alternatively, the two MRs may be sensitive to structurally distinct species. The decay profiles of DiSC_2_ and ThT are complex, requiring overall 2 or 3 decay components to fit the time-resolved traces adequately. While the τ_m_ values reveal an overall increase in viscosity of the aggregating sample and can help identify kinetic change, the species causing these changes cannot be determined. Neither can we rule out that very low concentrations of fibrillar species are causing these changes. To identify these species’ and distinguish an increase in τ_m_ due to oligomers or fibrils, phasor analysis was used.

### The Fluorescence Lifetime of DiSC_2_ and ThT Is Sensitive to Early Stage Aggregates

**Figure 2:**
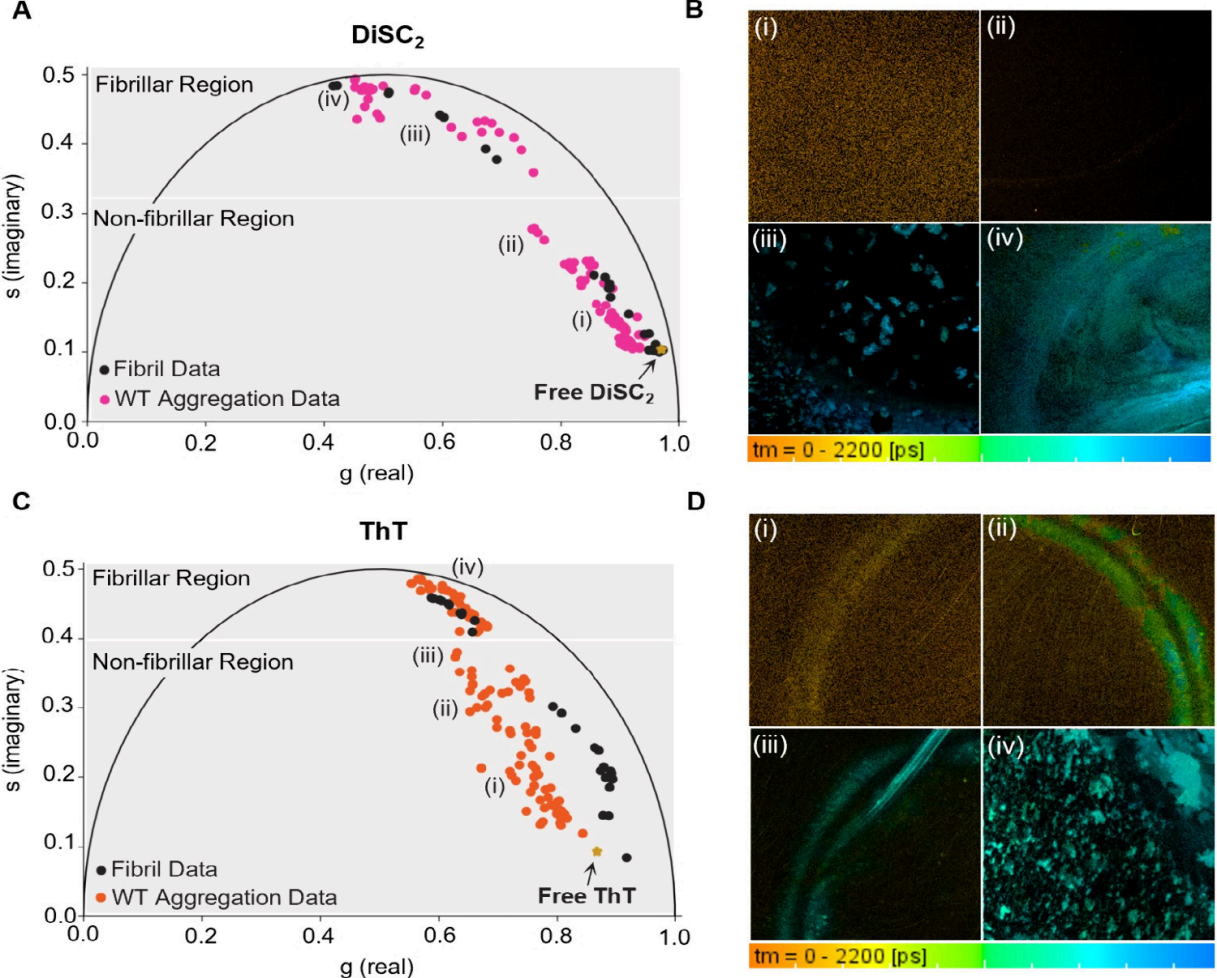
Phasor plots and FLIM images of WT αSyn aggregation monitored with DiSC_2_ and ThT in PBS. (A) Phasor analysis of DiSC_2_ (3 μM) in the presence of aggregating WT αSyn (150 μM, pink); data were combined from four DiSC_2_ repeats (Figure S4). Phasor analysis of DiSC_2_ (3 μM) in the presence of WT αSyn fibrils (0.001–150 μM, black) is overlaid (Figure S6). Any differences between the aggregation and the fibril plots indicate the presence and detection of nonfibrillar species in the aggregating solution. The fibrillar and oligomeric region highlighted in gray represent the regions in which the MRs are detecting specific species during the aggregation. Selection of the fibrillar and nonfibrillar regions (shown in gray) was based on the degree of overlap between the aggregation data and the fibrillar plot. Substantial overlap is considered when at least 60% of aggregation points in that region overlap with the fibrillar data. Importantly, they do not represent the only regions where these species reside on the phasor plot (see Figure S19 for further description). The time-resolved fluorescence decay of free DiSC_2_ can be found in Figure S14. (B) FLIM images of DiSC_2_ associated with the aggregation data set shown in panels A and B of Figure 1. The images were recorded at (i) 3 h, (ii) 5.5 h, (iii) 12 h, and (iv) 24 h. (C) Phasor analysis of ThT (10 μM) in the presence of aggregating WT αSyn (150 μM, orange); data combined from five ThT repeats (Figure S5). Phasor analysis of ThT (10 μM) in the presence of WT αSyn fibrils (0.001–150 μM, black) is additionally overlaid (Figure S7). Species specific regions, highlighted in gray, are assigned as described for DiSC_2_ (see Figure S20 for further description). The time-resolved fluorescence decay of free ThT can be found in Figure S15. (D) FLIM images of ThT associated with the aggregation data set shown in panels C and D of Figure 1. The images were recorded at (i) 2 h, (ii) 2.5 h, (iii) 3 h, and (iv) 27 h. It should be noted that, for both DiSC_2_ and ThT, depending on the aggregation repeat slight differences in the time frame can be observed.

Phasor plots represent a Fourier transform-based analysis which obtains a real (g) and imaginary (s) component of each fluorescence time-resolved decay associated with each time-point during an aggregation. This is a nonbiased analysis that does not require assumptions on the decay model, but instead, the shape of the phasor trajectory during aggregation can help assign the nature of different species detected. Points in a similar position on the universal semicircle likely correspond to a similar environment (monomeric, oligomeric and fibrillar) and points lying along a straight line are likely from the same environment or species but at a varying concentration.^18,27^ The individual phasor plots of DiSC_2_ and ThT from multiple repeats of monitoring WT αSyn aggregation were combined to create a full phasor plot (Figure 2A,C and Figures S4 and S5). In general, the phasor plots show a progression of points from the bottom right to the upper left of the universal semicircle, consistent with increased fluorescence lifetimes. Alongside aggregation, fibril phasor plots are shown in black, where increasing concentrations (0.001–150 μM) of preformed fibrils were measured in the presence of a fixed concentration of MRs (3 μM DiSC_2_ and 10 μM ThT). These phasor points represent confinement conditions created by fibrils alone. Thus, any differences between aggregation and fibril points indicate the detection of nonfibrillar species. However, the similarities in phasor points might mean either (i) that the points belong to fibrils present in the aggregation mixture, or (ii) that they coincide due to a similar environment created by oligomers and fibrils.

The DiSC_2_ phasor aggregation phasor plot (Figure 2A) can be separated into two distinct regions. The first region (*Nonfibrillar region*, Figure 2A,B) occurs early in the aggregation when monomeric and smaller aggregates are expected to dominate and where we observe a partial deviation from the fibril trajectory. This suggests we are detecting the presence of a species with a structure that differs from mature fibrils. The second region (*Fibrillar Region*, Figure 2A) occurs when aggregates at a size above the resolution limit of optical microscopy (300 nm) are observed in the lifetime images (Figure 2B), suggesting that fibrillar species are present and dominate the signal of the MR. This is supported by phasor analysis of DiSC_2_ lifetimes in the presence of increasing fibril concentrations, which shows a clear overlap of phasor coordinates at later aggregation times.

The individual aggregation monitored by DiSC_2_ in Figure 1B enters the fibrillar region of the phasor plot after 5.5 h (Figure 2A, ii to iii), this is associated with a substantial τ_m_ increase (Figure 1B). Interestingly, before 5.5 h, a small increase from 0.07 to 0.15 ns is observed (Figure 1B), which is associated with a movement of phasor points in the *Nonfibrillar Region* of the phasor plot (Figure 2A). The τ_m_ of DiSC_2_, therefore, does increase in the presence of oligomeric species; however, the most substantial τ_m_ increase occurs when the formation of fibrils begins.

The aggregation of WT αSyn monitored by ThT (Figure 2C,D) shows significant deviation from the fibril phasor plot in the early stages of the aggregation (*Nonfibrillar Region*, Figure 2C) and overlap only occurs later in the pathway (*Fibrillar Region*, Figure 2C). This suggests that ThT is sensitive to an early aggregate species which has a distinctly different structure to mature fibrils, likely oligomers.^28^

The individual aggregation monitored by ThT in Figure 1D enters the fibrillar region after 3 h (Figure 2C, iii to iv), therefore the initial increase in ThT τ_m_ observed can be unequivocally attributed to the presence of oligomeric species.

### DiSC_2_ and ThT Are Sensitive to the Presence of Structurally Distinct Oligomers

It has been reported that the aggregation pathway of WT αSyn involves the formation of two types of oligomers, called Type-A and Type-B.^28,29^ Type-A oligomers can be considered an early stage species while Type-B oligomers are a late-stage species which possess a higher β-sheet content compared to Type-A oligomers.^28^ We sought to determine whether the MRs were specific to either oligomer type. To do this, an enriched stabilized oligomer solution was prepared, where the oligomers have been shown to structurally resemble Type-B oligomers.^17^ In a similar manner to the fibril phasor plots, the lifetime decay traces of the MRs, at a constant concentration, were measured in the presence of increasing concentrations of stabilized oligomers.

**Figure 3:**
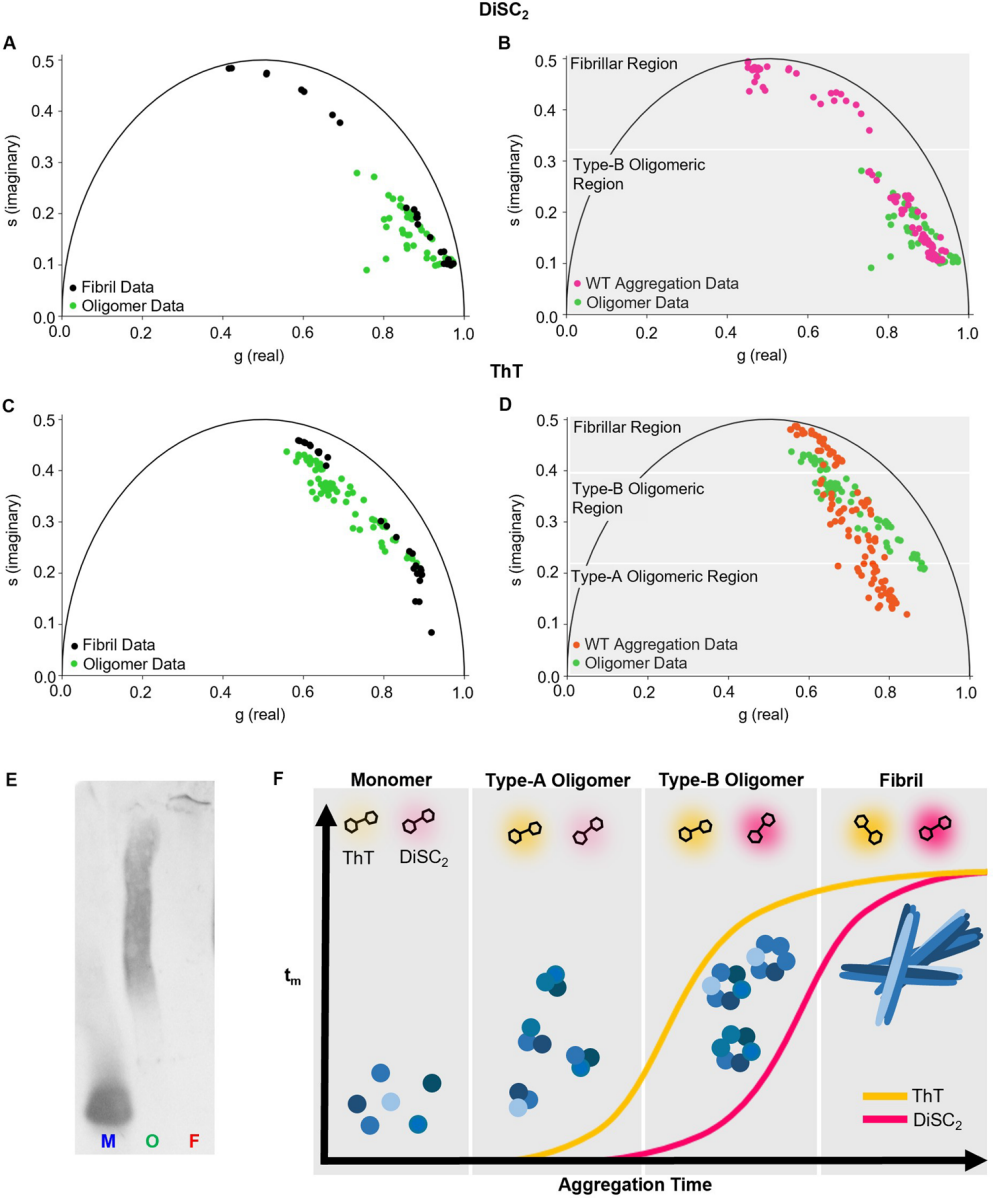
Comparative phasor analysis of time-resolved decay traces of DiSC_2_ and ThT in the presence of different WT αSyn species in PBS. (A) Phasor analysis of DiSC_2_ (3 μM) in the presence of isolated fibrils (0.001–50 μM, black) and stabilized oligomers (0.001–20 μM, green) (Figures S6 and S8). (B) Phasor analysis of DiSC_2_ (3 μM) in the presence of aggregating WT αSyn (150 μM, pink) as shown in Figure 2A and stabilized oligomers (0.001–20 μM, green) (Figures S4 and S8). (C) Phasor analysis of ThT (10 μM) in the presence of isolated fibrils (0.001–150 μM, black) and stabilized oligomers (0.001–50 μM, green) (Figures S7 and S9). (D) Phasor analysis of ThT (10 μM) in the presence of aggregating WT αSyn (150 μM, orange) as shown in Figure 2C and stabilized oligomers (0.001–50 μM, green) (Figures S5 and S9). The fibrillar and oligomeric (Type-A and Type-B) regions highlighted in gray represent the regions in which the MRs are detecting specific species during the aggregation. Selection of the gray regions was based on the degree of overlap between the aggregation data, the fibrillar plot, and the stabilized oligomeric plot. Substantial overlap is considered when at least 60% of aggregation points in that region overlap with the fibrillar or stabilized oligomer data. Importantly, they do not represent the only regions where these species reside on the phasor plot (see Figures S19 and S20 for further description). (E) Native-PAGE and Western blot analysis of monomeric (M), oligomeric (O), and fibrillar (F) WT αSyn [see also dynamic light scattering (DLS) analysis in Figure S21]. (F) Schematic diagram of the changes in τ_m_ of DiSC_2_ and ThT during the aggregation of WT αSyn, with the dominant species stated at each stage.

The stabilized oligomers detected by DiSC_2_ reside at the bottom right of the phasor plot (Figure 3A). These points show a partial overlap with the fibril phasor points, which could be due to the fact that both stabilized oligomers and fibrils have significant β-sheet content. Importantly, the early time-points of WT αSyn aggregation show a much better overlap with the stabilized oligomer phasor points than with the fibril phasor points (Figure 3B). We conclude that the early stage aggregates sensed by DiSC_2_ are structurally similar to the stabilized oligomers, *i.e*., Type-B oligomers. We define the region of the phasor plot where the points from the aggregation of WT αSyn and the stabilized oligomers overlap as the *Type-B Oligomeric Region* (Figure 3B). These results also suggest that the initial increase in τ_m_ described in Figure 1 is probably due to the formation of Type-B oligomers.

Similarly, we observed an overlap between WT αSyn aggregation phasor points and stabilized oligomers detected by ThT (Figure 3D). This suggests that ThT also senses aggregates which are structurally similar to stabilized oligomers, *i.e*., Type-B oligomers, and in this case, it is again possible to define a phasor plot region as the *Type-B Oligomeric Region*. The fact that Type-B oligomers can be sensed by ThT explains the substantial τ_m_ increase, from 0.07 to 0.45 ns, observed between 2 and 3 h in Figures 1D and 2C (i and iii). However, at very early time-points, there is a clear deviation of the WT αSyn aggregation phasor plot from both the stabilized oligomer and fibril phasor points detected by ThT (Figure 3D). These phasor points reflect an initial increase in τ_m_, from 0.012 to 0.7 ns, between 0 and 2 h in Figures 1D and 2C (i). This observation suggests that the ThT τ_m_ can initially sense the formation of aggregates which are structurally distinct from both stabilized oligomers and fibrils. This species may be Type-A oligomers, which are known to have a low β-sheet content,^17^ likely causing the large deviation from the other phasor plots. For this reason, we define this region of the phasor plot as the *Type-A Oligomeric Region*. Altogether, these results suggest that ThT can report on both the presence of Type-A and Type-B oligomers with the τ_m_ showing a large increase in the presence of Type-B oligomers compared to Type-A oligomers.

We have shown that DiSC_2_ and ThT can be used to monitor the aggregation of WT αSyn. Both MRs show an increase in τ_m_ which can be attributed to the presence of oligomers. Importantly, for both DiSC_2_ and ThT, phasor analysis can be utilized to identify the formation of oligomeric species (Type-A and Type-B for ThT, and Type-B for DiSC_2_), even if the τ_m_ does not show a substantial increase.

### The ThT Lifetime Confirms That the A30P αSyn Variant Shows Delayed Oligomer-Fibril Conversion

**Figure 4:**
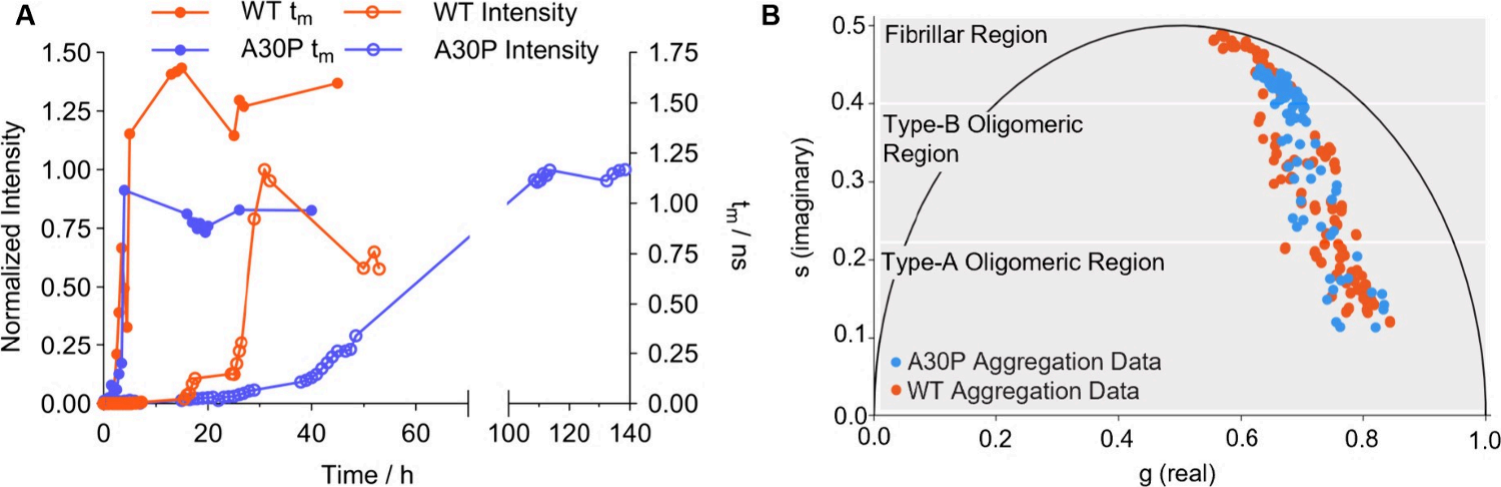
Lifetime analysis and comparative phasor plots of A30P and WT αSyn aggregation monitored with ThT in PBS. (A) Comparison of the intensity (empty circles) and τ_m_ (filled circles) of ThT (10 μM) in the presence of A30P (150 μM, blue) and WT αSyn (150 μM, orange) during a single aggregation repeat; the full data set from all repeats is shown in Figures S5 and S10. (B) Phasor analysis of the time-resolved decay traces of ThT (10 μM) in the presence of aggregating A30P αSyn (blue, data combined from three repeats, as shown in Figure S10), overlaid with the data for WT αSyn (orange, data from Figure 2).

As shown above, ThT can report on both the presence of Type-A and Type-B oligomers while DiSC_2_ is sensitive to only Type-B (Figure 2). ThT was therefore used to monitor the aggregation of two further variants which have been reported to have different aggregation kinetics with respect to WT αSyn. First, we investigated the behavior of the genetic mutant A30P αSyn (Figure 4 and Figures S2, S10, and S11), which has been reported to aggregate slower than WT αSyn and enable a buildup of oligomers.^30,31^

A substantial increase in ThT τ_m_ is observed after 2 h in the presence of aggregating WT and A30P αSyn (Figure 4A) but the lag phase recorded by ThT intensity is extended for A30P αSyn compared to WT αSyn (Figure 4A). This suggests that the rate of Type-B oligomer formation is similar for the two proteins, but the rate of oligomer conversion into fibrils is slower for A30P αSyn. Our results support previous studies which show that A30P αSyn causes an accumulation of oligomers compared to WT αSyn.^30,31^

The final lifetime values seen for A30P αSyn are also lower compared to WT αSyn. This could reflect a different structure of species present (both oligomers and fibrils), or a different mode of interaction of ThT with these protein species.

There are small but significant differences between the aggregation phasors of WT and A30P αSyn at time-points collected both at early and late stages of aggregation (Figure 4B). These differences indicate potential structural differences between the oligomers of A30P and WT αSyn. However, given that A30P αSyn has been reported to accumulate oligomers,^31^ we cannot exclude that these differences are due to a higher concentration of A30P αSyn oligomers. In fact, increasing concentration of a protein species could cause a proportional shift in the lifetime.

### The ΔP1 αSyn Variant Delays Formation of Oligomeric Species during Aggregation

**Figure 5:**
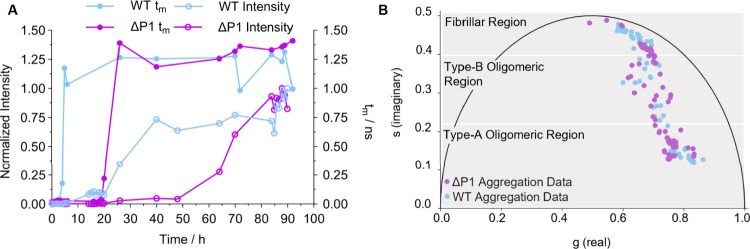
Lifetime analysis and comparative phasor plots of ΔP1 and WT αSyn aggregation monitored with ThT in 20 mM Tris-HCl with 200 mM NaCl. (A) Comparison of the intensity (empty circles) and τ_m_ (filled circles) of ThT (10 μM) in the presence of ΔP1 (100 μM, purple) and WT αSyn (100 μM, light blue) during a single aggregation repeat; the full data set from all repeats is shown in Figures S12 and S13. (B) Phasor analysis of the time-resolved decay traces of ThT (10 μM) in the presence of aggregating ΔP1 αSyn (purple, data combined from three repeats as shown in Figure S13), overlaid with the data from the aggregation of WT αSyn (light blue, data combined from three repeats as shown in Figure S12).

The “P1 region” of αSyn (residues 36–42) has recently been identified as a master regulator of amyloid aggregation.^32,33^ In fact, a variant of αSyn missing this protein region, *i.e*., ΔP1 αSyn, has been shown to accumulate as oligomers, resulting in reduced amyloid formation.^32,33^ We set out to test whether our method could uncover further details about the time evolution of the oligomers of this protein (Figure 5 and Figures S3, S12, and S13). We found that, for WT αSyn, ThT τ_m_ increased rapidly after 3 h, whereas for ΔP1 αSyn, this increase was observed after ∼20 h (Figure 5A). This observation suggests that the deletion of the P1 region delays the formation of oligomers. The increase in ThT intensity was also delayed, t_50_ ∼ 70 h for ΔP1 αSyn vs ∼30 h for WT αSyn (Figure 5A). This suggests that the P1 region plays a key role in regulating the rate of both oligomer and fibril formation. Interestingly, the deletion of the P1 region causes a greater delay in the initial ThT intensity increase (+ ∼ 40 h) compared to the initial lifetime increase (+ ∼ 17 h), relative to WT αSyn. This suggests that the conversion of oligomers to fibrils is more significantly impacted than the initial oligomer formation. This supports a recent study showing that the P1 region acts as a controller for the conversion of oligomers to fibrils.^34^

The same final τ_m_ value (∼1.5 ns) (Figure 5A) was reached for WT αSyn and ΔP1 αSyn, this indicates a similar degree of crowding of ThT in both variants and supports previous literature which suggests that the morphologies of the mature amyloid fibrils are similar.^32,34^ Furthermore, both WT and ΔP1 αSyn aggregation phasor trajectories show a good degree of overlap (Figure 5B), suggesting similar structural interaction with ThT (*i.e*. similar proportions of species present) enroute to fibrils, albeit at very different kinetics (Figure 5A).^32,34^

## Discussion

We have developed a method based on the combined use of fluorescence lifetime and intensity of two MRs, ThT and DiSC_2_,^18,24^ to monitor the aggregation of WT αSyn and its variants, in real time, including the formation of oligomers.

We found that the τ_m_ of both MRs was more sensitive to changes in the aggregation of WT αSyn compared to intensity (Figure 1B,D). However, τ_m_ analysis alone could not help us identify the structural species present in the aggregating solution. For this, we utilized phasor analysis; a nonbiased fitting analysis of individual time-resolved decays. We observed that the aggregation phasor plots of both MRs showed deviation from isolated fibril phasors indicating their ability to sense an early stage aggregate, most likely oligomers (Figure 2A,C).

To further characterize these early stage aggregates, we prepared an enriched stabilized oligomer solution, where the oligomers have been reported to possess a structure similar to Type-B oligomers.^17^ These Type-B oligomers are a class of late-stage oligomers which have a β-sheet content similar to mature fibrils and are highly toxic.^28^ Type-A oligomers, the other main class of oligomers that form during an *in vitro α*Syn aggregation, form early in the aggregation and are considered weakly toxic.^28,29^

Phasor analysis of the time-resolved decay traces of DiSC_2_ in the presence of stabilized oligomers showed a clear overlap with the early stages of the aggregation phasor (*Type-B Oligomeric Region*, Figure 3B). These data suggest that DiSC_2_ can be used to probe Type-B oligomers, however the τ_m_ only begins to significantly increase when the phasor enters the *Fibrillar Region* (Figures 1B and 3B).

Phasor analysis of the time-resolved decay traces of ThT in the presence of stabilized oligomers showed significant overlap with some of the aggregation phasor (*Type-B Oligomeric Region*, Figure 3D), indicating ThT is also sensitive to the presence Type-B oligomers. Moreover, a key region of the phasor plot was identified where there is no overlap of the aggregation phasor points associated with the very early stages of aggregation and either the stabilized oligomer or fibril phasor plots (*Type-A Oligomeric Region*, Figure 3D). Therefore, ThT can detect the presence of an early stage oligomeric species which is structurally distinct from stabilized oligomers. These species are most likely Type-A oligomers. Importantly, a significant increase in τ_m_ is only observed when the *Type-B Oligomeric Region* (Figures 1D and 3D) is entered, indicating that the kinetic characterization of Type-B oligomers can be achieved using ThT.

Finally, we used our approach to monitor the oligomer formation of two variants of αSyn, named A30P and ΔP1 αSyn, using ThT. We validated our method on these two proteins because they present major differences in the aggregation behavior with respect to WT αSyn.^30,32,34^ For A30P αSyn, we found that the ThT τ_m_ increased at a similar rate to that of WT αSyn during aggregation. This observation suggests that the rate of oligomer formation of A30P αSyn is comparable to that of WT αSyn. Conversely, ThT intensity of A30P αSyn increased at a significantly lower rate. This result indicates that the oligomer conversion into fibrils of A30P αSyn is significantly delayed with respect to that of WT αSyn. The similar rate of formation of oligomer, but delayed conversion of oligomers into fibrils could explain the accumulation of oligomers observed for A30P αSyn.^30,31^

In contrast, both ThT τ_m_ and intensity increases were delayed for ΔP1 αSyn compared to WT αSyn. This result suggests that the P1 region plays a role in regulating both oligomer and fibril formation. Nevertheless, the effect on ThT intensity was greater than that on ThT τ_m_, indicating that that deletion of the P1 region predominantly affects the conversion of oligomers into fibrils rather than the initial formation of the oligomers. These findings were in agreement with previous literature^30,34^

## Conclusions

Our molecular rotor-based approach provides an exciting toolset for monitoring the aggregation of αSyn and provides structural insight into the aggregated species formed by the protein. The sensitivity of this approach for the oligomers demonstrates its high potential in comparison to standard bulk-averaged intensity measurements. By using fluorescence intensity and lifetime in combination, we can unveil the complicated mechanism of αSyn amyloid formation to identify and monitor key targets in PD pathology, therefore, opening doors to improving diagnostic and therapeutic strategies.

## Materials and Methods

### Expression and Purification of WT αSyn

BL21-Gold (DE3) competent *Escherichia coli* cells (Agilent Technologies, Santa Clara, CA, USA) were transformed with the plasmid pT7–7 WT αSyn (a gift from Hilal Lashuel, Addgene^35^) as per the manufacturer’s instructions and used to express WT αSyn. The silent mutation TAC136TAT was introduced into the WT αSyn DNA sequence to prevent Cys misincorporation and dimerization.^36^ Expression was scaled up to 4 L and carried out at 37 °C for 4 h. To harvest the cells, the suspensions were centrifuged and resuspended in 20 mM Tris-HCl, 1 mM EDTA, pH 8.0, including 1 tablet/2 L of bacterial culture of cOmplete, Mini, EDTA-free Protease Inhibitor Cocktail (Roche, Basel, Switzerland). The subsequent purification of WT αSyn was then carried out as previously described.^37^ The lysate was sonicated on ice and the supernatant, following centrifugation (30 min, 18,000 rpm, 4 °C) was boiled at 80 °C for 20 min to precipitate heat-sensitive proteins. The denatured protein was removed by centrifugation (30 min, 18,000 rpm, 4 °C) and the supernatant was incubated with streptomycin sulfate, (20 mg/mL, 20 min, 4 °C) to precipitate the DNA which was subsequently removed by centrifugation (30 min, 18,000 rpm, 4 °C). The slow addition of ammonium sulfate (360 mg/mL, 20 min, 4 °C) while stirring precipitated out the αSyn which was collected by centrifugation and the pellet was resuspended in 20 mM Tris-HCl, 1 mM EDTA, pH 8.0. To ensure complete buffer exchange, dialysis of the resuspended sample was carried out overnight at 4 °C. The protein solution was then purified by ion-exchange chromatography (IEX) using a HiPrep Q HP 16/10 column (Cytiva Life Sciences, Marlborough, MA, USA) and the eluted protein was subsequently purified by size-exclusion chromatography (SEC) using a HiLoad 16/600 Superdex 75 pg column (Cytiva Life Sciences, Marlborough, MA, USA). The purified protein was eluted in PBS (100 mM Na2HPO4, 18 mM KH2PO4, 1.37 M NaCl, 27 mM KCl, pH 7.4). The final purified protein concentration was determined by UV–vis spectroscopy using a Cary 60 UV–vis spectrometer (Agilent Technologies, Santa Clara, CA, USA) by recording the absorbance at 275 nm and a molar extinction coefficient of 5600 M^–1^ cm^–1^. The purity of the protein was assessed using time-of-flight mass spectrometry with electrospray ionization (ESI-TOF MS) and sodium dodecyl sulfate–polyacrylamide gel electrophoresis (SDS-PAGE). ESI-TOF MS was performed at the Chemistry Mass Spectrometry facilities of the Molecular Sciences Research Hub, Department of Chemistry, Imperial College London (Figure S23). SDS-PAGE was performed using a NuPAGE Bis-Tris gel (4−12%, 1.0−1.5 mm; Thermo Fisher Scientific, Waltham, MA, USA) following the manufacturer’s instructions (Figure S26).

### Expression and Purification of αSyn Variants (A30P and ΔP1 αSyn)

The A30P αSyn plasmid was prepared by site-directed mutagenesis of the pT7–7 WT αSyn plasmid using a QuikChange II Site-Directed Mutagenesis Kit (Agilent, Agilent Technologies, Santa Clara, CA, USA), the primers (obtained from Eurofins Scientific, Luxembourg City, Luxembourg) are given in Table 1.

**Table 1:** A30P αSyn Variant Primer Sequences^a^

	sequence (5′–3′)	*T*_m_ (°C)
reverse primer	tcttttgtctttcctggtgcttctgccacaccc	79.1
forward primer	gggtgtggcagaagcaccaggaaagacaaaaga	79.1

a Sequences of the forward and reverse primers for the mutagenesis of the pT7-7 WT αSyn plasmid to produce the A30P αSyn variant plasmid.

The ΔP1 αSyn plasmid was obtained from Professor Sheena E. Radford (University of Leeds). Both variants were then transformed and expressed following the same method as described above for WT αSyn. The concentration of the variants was determined by UV–vis spectroscopy using a molar extinction coefficient of 5600 M^–1^ cm^–1^ for A30P αSyn and 4470 M^–1^ cm^–1^ for ΔP1 αSyn, at an absorbance of 275 nm. The variants were stored in PBS (pH 7.4). For the aggregations of ΔP1 αSyn the variant was buffer exchanged into 20 mM Tris-HCl with 200 mM NaCl (pH 7.5) or 20 mM sodium acetate with 200 mM NaCl (pH 4.5). As for WT αSyn, ESI-TOF MS and SDS-PAGE was used to assess the purity of the protein (Figures S24–S26).

### Preparation of αSyn Fibrils

αSyn fibrils were prepared by incubating solutions of monomeric αSyn (∼200 μM) at 37 °C for 5 days with agitation (600 rpm). The resulting fibrils were pelleted by centrifugation (14,000 rpm) and resuspended in the required buffer. The centrifugation and resuspension steps were repeated three times. The final fibril concentration, following treatment with guanidinium hydrochloride (Gnd-HCl) to a final concentration of 4 M, was estimated using UV–vis spectroscopy by recording the absorbance at 275 nm and a molar extinction coefficient of 5600 M^–1^ cm^–1^. This gave the concentration in monomer equivalents. See the Supporting Information for DLS characterization (Figure S21) and negative stain transmission electron microscopy (TEM) imaging (Figure S22).

### Preparation of αSyn Stabilized Oligomers

An enriched solution of αSyn stabilized oligomers was prepared using an adapted protocol from Chen et al.^17^ 400 μL of 800 μM monomeric αSyn in PBS (pH 7.4) was flash frozen in liquid nitrogen and lyophilized overnight. The lyophilized protein was resuspended in PBS (pH 7.4) and incubated at 37 °C for 24 h without agitation to promote the formation of oligomeric species. Fibrillar species were then removed by ultracentrifugation (55,000 rpm, 1.25 h) and the monomeric protein removed by multiple filtration steps using a 100 kDa cutoff membrane. The final oligomeric solution concentration was estimated by UV–vis spectroscopy using a nanodrop at 275 nm and a molar extinction coefficient of 7000 M^–1^ cm^–1^.

### Native-PAGE and Western Blotting

Monomeric, oligomeric and fibrillar samples were prepared as previously described. Each sample (20 μL) was added to 20 μL native sample buffer and ran on a Novex Tris-Glycine gel (Thermo Fisher Scientific, Waltham, MA, USA) in native running buffer, following the manufacturer’s instructions. An iBlot 2 (Thermo Fisher Scientific, Waltham, MA, USA) was used to transfer the gel onto a 0.45 μM nitrocellulose (7 min, 25 V). The membrane was blocked in 4% nonfat milk in PBS-T (PBS + 0.1% Tween) for 1 h at room temperature (RT). Following washing with PBS-T, the membrane was incubated in anti-α-syn primary antibody [MJFR1] (Abcam, Cambridge, UK) at a 1:1000 dilution in PBS-T for 1 h at RT. The membrane was washed in PBS-T (2 × 10 min, RT) and then incubated with Alexa Fluor 555 goat antirabbit secondary antibody (Thermo Fisher Scientific, Waltham, MA, USA) at a 1:5000 dilution in PBS-T, at RT for 1 h, protected against light. Following further washing with PBS-T (2 × 10 min, RT), the membrane was detected using a 555 nm laser using a Typhoon FLA 9500 scanner (GE Healthcare, Amersham, UK).

### Dynamic Light Scattering

Monomeric, oligomeric and fibrillar samples were prepared as previously described. DLS of the samples (∼10 μM, 100 μL) in PBS (pH 7.4) were measured using a Malvern Zetasizer Ultra (Malvern Panalytical, Malvern, UK), in low-volume disposable cuvettes.

### Negative Stain Transmission Electron Microscopy

Fibrillar samples at the end point of aggregation (4 μL) were spotted on Formvar/carbon-coated 300 mesh copper grids for 1 min. Whatman filter paper was used to blot the excess sample from the grid and then allowed to dry for a further 2 min. The grids were then washed with water (4 μL) and stained with 2% w/v uranyl acetate. A T12 Spirit electron microscope (Thermo Fisher Scientific (FEI), Hillsboro, OR, USA) was used to image the grids.

### Amyloid Formation Monitored by Fluorescence Intensity and Fluorescence Lifetime Imaging

WT and A30P αSyn (150 μM) monomer solutions were aggregated in the presence of 10 μM ThT or 3 μM DiSC_2_, 0.02% sodium azide (NaN_3_) and PBS (pH 7.4). 120 μL of each sample was incubated in a 96 well full-area glass bottom plate (Sensoplate, Greiner Bio One, UK) with a borosilicate bead (3 mm diameter) at 37 °C for 24–48 h in a benchtop incubator, shaking at 450 rpm. The plate was covered with a clear film (ibiSeal, Ibidi, Germany). At specific incubation times, the intensity and fluorescence lifetime were measured.

ΔP1 and WT αSyn (100 μM) monomer solutions were aggregated in the presence of 10 μM ThT, 0.02% NaN_3_ and 20 mM Tris-HCl with 200 mM NaCl (pH 7.5) or 20 mM sodium acetate with 200 mM NaCl (pH 4.5). 100 μL of each sample was incubated in a 96 well full-area glass bottomed plate at 37 °C for ∼60 h in a benchtop incubator, shaking at 600 rpm. The plate was covered with a clear film. At specific incubation times, the intensity and fluorescence lifetime were measured.

### Fluorescence Intensity Measurements

Fluorescence intensity measurements of the same samples measured by FLIM in the 96 well full-area glass bottomed plate were taken in a CLARIOstar Plus microplate reader (BMG Labtech, Ortenberg, Germany). Spiral averaging (3 mm diameter), excitation 440 nm, dichroic 460 nm, and emission 480 nm filters (ThT) or excitation 530 nm, dichroic 552.5 nm and emission 580 nm (DiSC_2_), 4 gains and 50 flashes per well was used. Aluminum adhesive seals were used on top of the clear film to cover the samples for the intensity measurements.

### Fluorescence Lifetime Imaging

The fluorescence lifetime images of samples on a borosilicate glass slide or 96 well full-area glass bottomed plate were 256×256 pixels and obtained using a Leica TSC SP5 II inverted confocal microscope (Leica Microsystems GmbH, Germany) with a TCSPC SPC830 single photon counting card (Becker & Hickl GmbH) and a 10x air objective (Leica Microsystem Ltd, Germany). Internal FLIM detector PMH-100 (Becker&Hickl, Germany), synchronised to a Ti:sapphire pulsed laser source (680−1080 nm, 80 MHz, 140 fs, Chameleon Vision II, Coherent Inc., Germany). A two-photon pulsed excitation (λ_ex_ = 880 nm for ThT and λ_ex_ = 950 nm for DiSC_2_) was used. Emission was recorded with an open pinhole from 465 to 540 nm for ThT and from 525 to 700 nm for DiSC_2_. For each sample a fluorescence lifetime image at least two *z*-positions was measured. The IRF used for deconvolution was recorded using the reflection of the excitation beam from crystals of urea, grown on the glass cover slide. The individual pixel decays were summed for each image and processed as described below.

### Fluorescence Lifetime Data Analysis

The equation below (eq 1) was used to fit the fluorescence decays to a mono-, bi- or triexponential model.
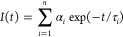
1where *I* is fluorescence intensity, *t* is time, and τ_*i*_ and α_*i*_ are the fluorescence lifetimes and amplitudes of the *n* exponentially decaying components, respectively. SPCImage (Becker & Hickl GmbH, Germany) was used to fit the data. For the time-resolved decays that fit the bi- or triexponential models, the amplitude-weighted mean lifetime (τ_*m*_) was also determined following the equation below (eq 2).
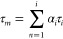
2

GraphPad Prism version 9.3.1 for Windows (GraphPad Software, San Diego, CA, USA) was used to plot the data. Phasor analysis was achieved using software written in-house in Matlab (MathWorks) and was performed on the time-resolved fluorescence decays of DiSC_2_ and ThT in the presence of the monomers and aggregates of WT, A30P and ΔP1 αSyn. By plotting the real (g) and imaginary (s) components of the Fourier transform of a fluorescence decay, a two-dimensional representation of the data can be obtained. The two components are calculated as shown below.
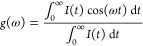
3
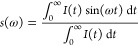
4where ω is the angular repetition frequency of the pulsed excitation laser (80 MHz), *I*(*t*) is the measured fluorescence decay and *t* is time. The phasors of the decays can be superimposed on a “universal circle” (central coordinates (1/2, 0)). For homogeneous systems in which only one fluorescence lifetime, τ, exists, the phasor points *g*(ω) = 1/[1 + (*ωτ*)^2^] (real) and *s*(ω) = *ωτ*/[1 + (*ωτ*)^2^] (imaginary) lie on the universal circle. This type of decay can be considered monoexponential, whereas phasors points of multiexponential decays will lie within this circle.^18^ Phasor analysis can therefore give an indication of the number of different species which form during the aggregation of αSyn.

### Statistical Analysis

GraphPad Prism software was used for all statistical analysis. To determine the regions of overlap in the phasor plots of DiSC_2_ and ThT in the presence of aggregating αSyn, stabilized oligomeric αSyn and fibrillar αSyn, error bars were applied to each phasor point. Error bars represent the standard deviation (2σ) of technical repeats of FLIM fibril and oligomer measurements. Two points were considered overlapping when there was overlap in two error bar directions. Regions were assigned if there was at least a 60% overlap between points.

## Data Availability

Source data for all main text and SI figures can be found at https://zenodo.org/records/13951260.

## Supporting Information Available

Intensity aggregation assays, individual lifetime repeat data and associated phasor plots, and structural analysis of the αSyn monomer, oligomers, and fibrils using DLS, EM, and MS (PDF)
