# Supplementary material for: Molecular Rotors Detect the Formation and Conversion of α-Synuclein Oligomers
Source: ACS Appl Mater Interfaces. 2025 Feb 5;17(7):10499–508. doi: 10.1021/acsami.4c21710 (PMC11843532; doi:10.1021/acsami.4c21710)
Supplement: Supplementary file 1 — am4c21710_si_001.pdf [file am4c21710_si_001.pdf]

Supporting Information for

Molecular Rotors Detect the Formation and Conversion of α-Synuclein Oligomers

Siân C. Allerton,[1,2] Marina K. Kuimova,[1,2] Francesco A. Aprile[1,2]**

[1]Department of Chemistry, Molecular Sciences Research Hub, Imperial College London, London W12 0BZ, UK.

[2]Institute of Chemical Biology, Molecular Sciences Research Hub, Imperial College London, London W12 0BZ, UK.

*To whom correspondence should be addressed: m.kuimova@imperial.ac.uk, f.aprile@imperial.ac.uk

This PDF file includes:

Figures. S1 to S26

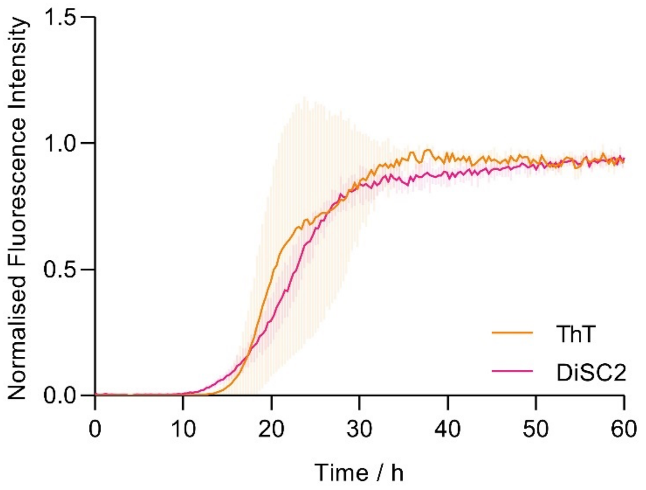

Figure S1. Aggregation of WT αSyn. Fluorescence intensity assay of WT αSyn (150 μM) in PBS (pH 7.4), monitored by $DiSC_2$ (3 μM) or ThT (10 μM). Done in triplicate.

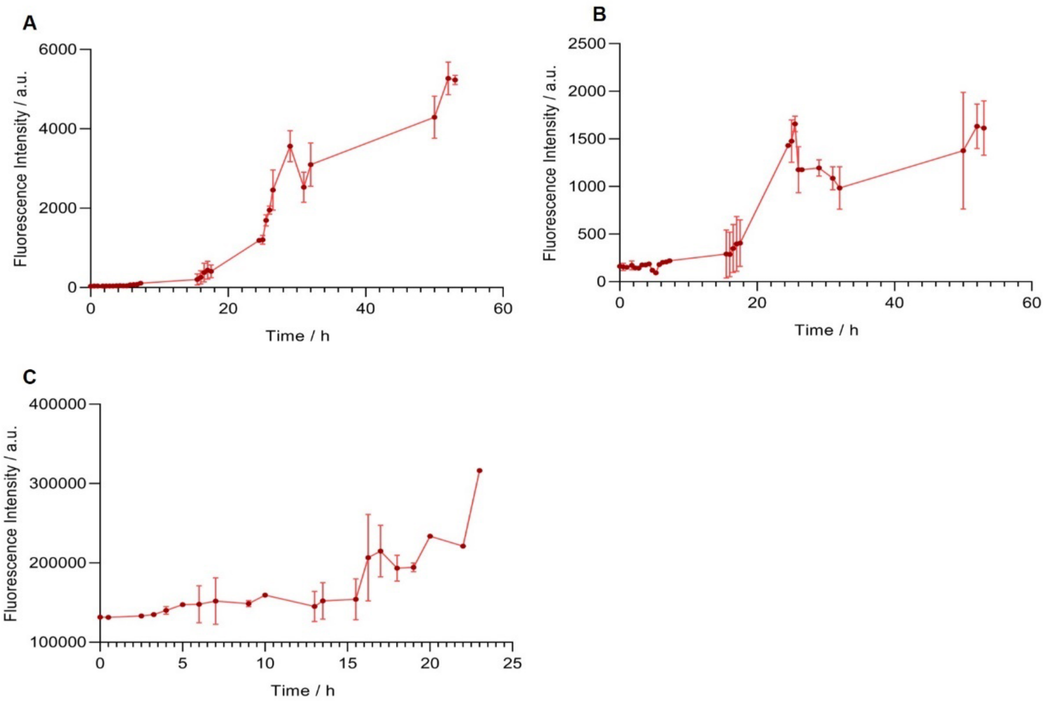

Figure S2. Aggregation of WT and A30P αSyn. (A) Fluorescence intensity assay of WT αSyn (150 µM) in PBS (pH 7.4), monitored by ThT (10 µM). (B) Fluorescence intensity assay of WT αSyn (150 µM) in PBS (pH 7.4), monitored by $DiSC_2$ (3 µM). (C) Fluorescence intensity assay of A30P αSyn (150 µM) in PBS (pH 7.4), monitored by ThT (10 µM). Done in duplicate.

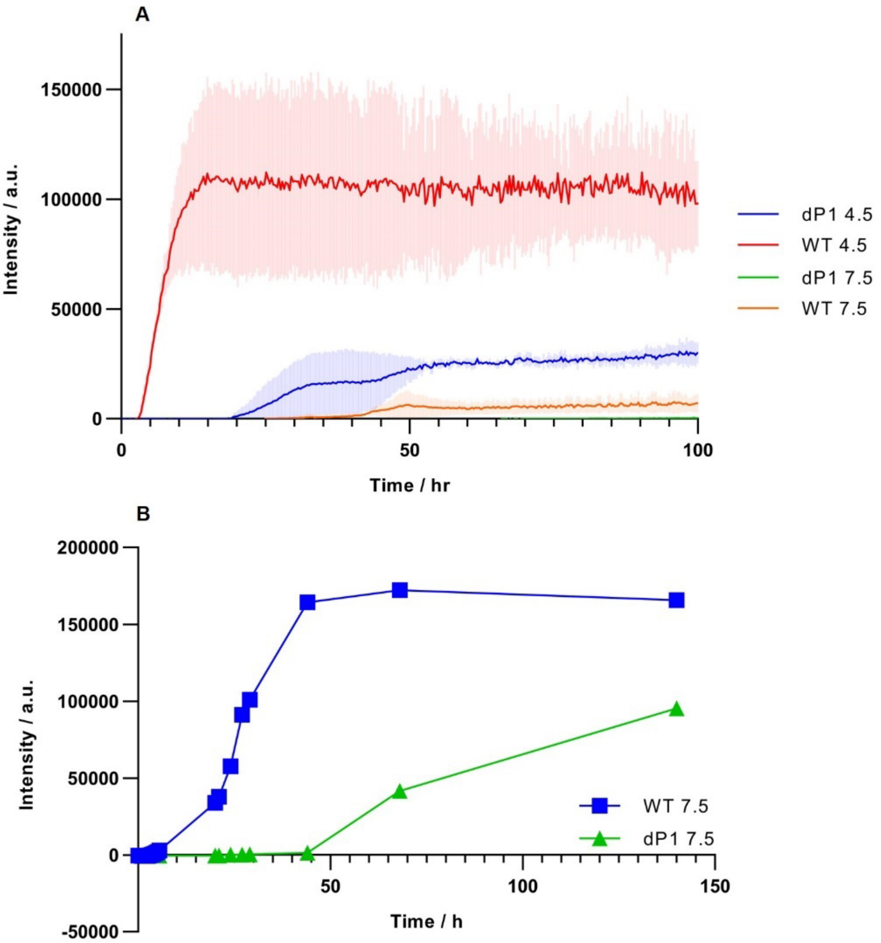

Figure S3. Aggregation of WT and ΔP1 αSyn. (A) Fluorescence intensity assay of WT and ΔP1 αSyn (100 μM) in 20 mM Tris-HCl with 200 mM NaCl (pH 7.5) or 20 mM sodium acetate with 200 mM NaCl (pH 4.5), monitored by ThT (10 μM). Done in triplicate. (B) Fluorescence intensity assay of WT and ΔP1 αSyn (100 μM) in 20 mM Tris-HCl with 200 mM NaCl (pH 7.5) monitored by ThT (10 μM).

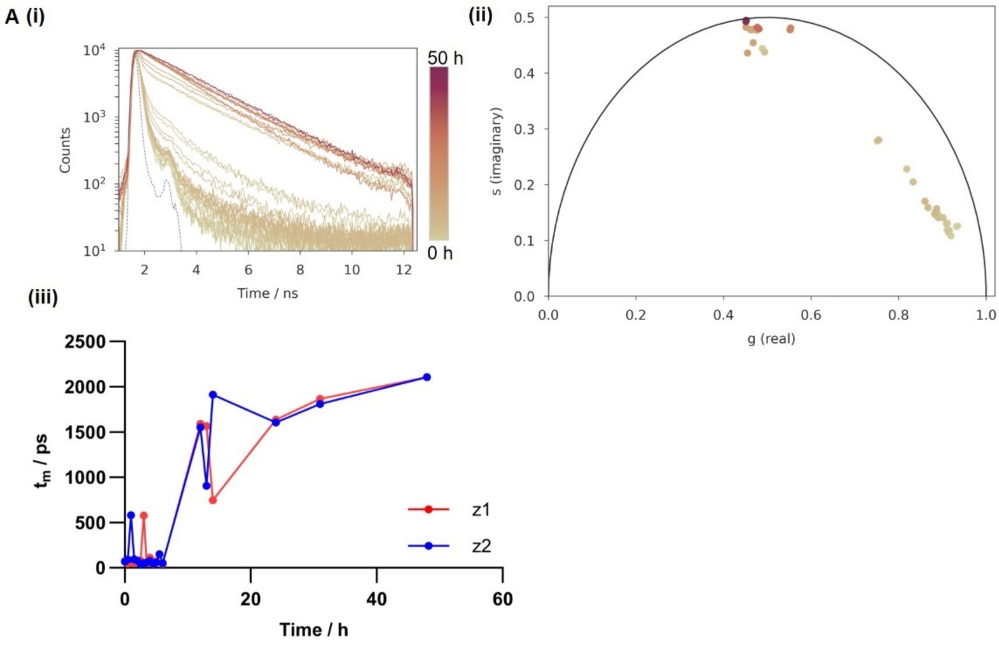
A (i)
(ii)
(iii)
Counts
Time / ns
50 h
0 h
s (imaginary)
g (real)
t_m / ps
Time / h
z1
z2

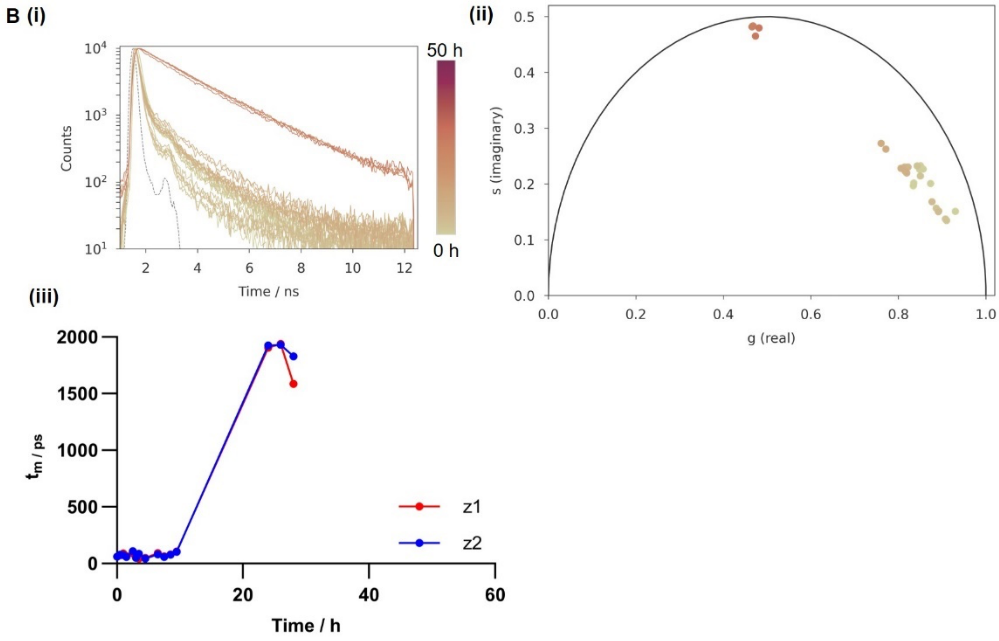
B (i)
(ii)
(iii)
Counts
Time / ns
50 h
0 h
s (imaginary)
g (real)
t_m / ps
Time / h
z1
z2

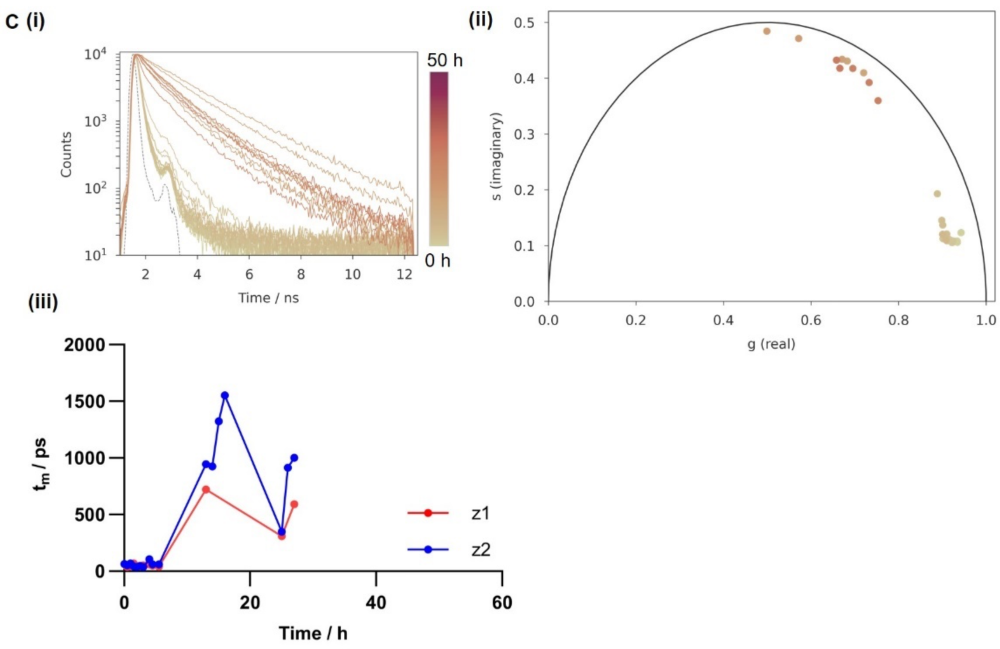

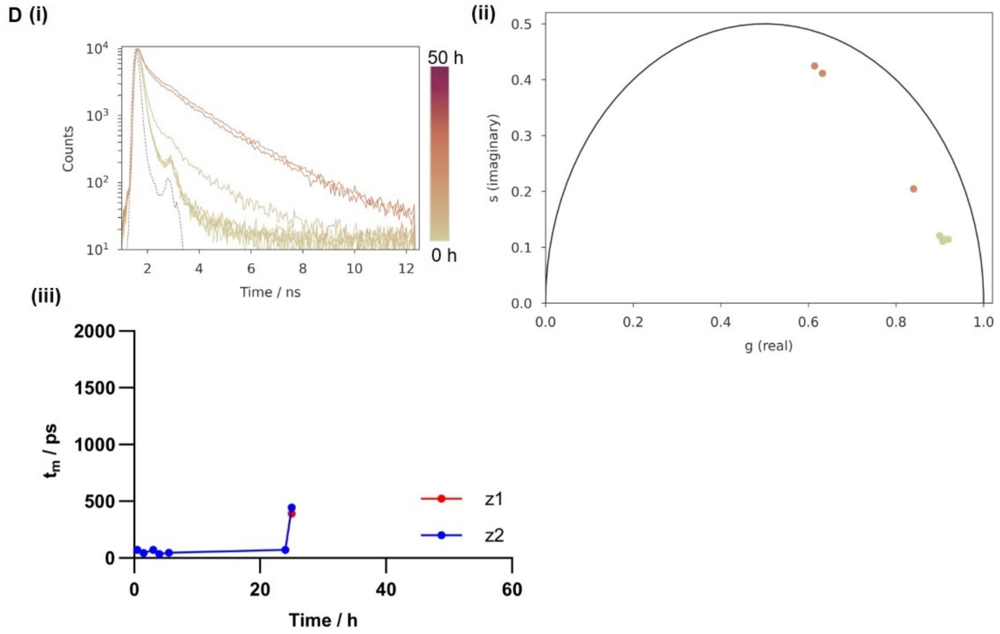

Figure S4. Repeats of $DiSC_2$ time-resolved fluorescence decays and lifetime analysis used to monitor WT αSyn aggregation in PBS (pH 7.4) (A–D). (i) Time-resolved fluorescence decays of $DiSC_2$ (3 µM) in the presence of aggregating WT αSyn (150 µM). (ii) Phasor analysis of $DiSC_2$ decay profile. (iii) Fitted lifetimes (τ_m) of $DiSC_2$ during the aggregation. z-positions were taken at ~ 100 nm above the well plate surface (z2) and ~ 1000 nm above this position (z1).

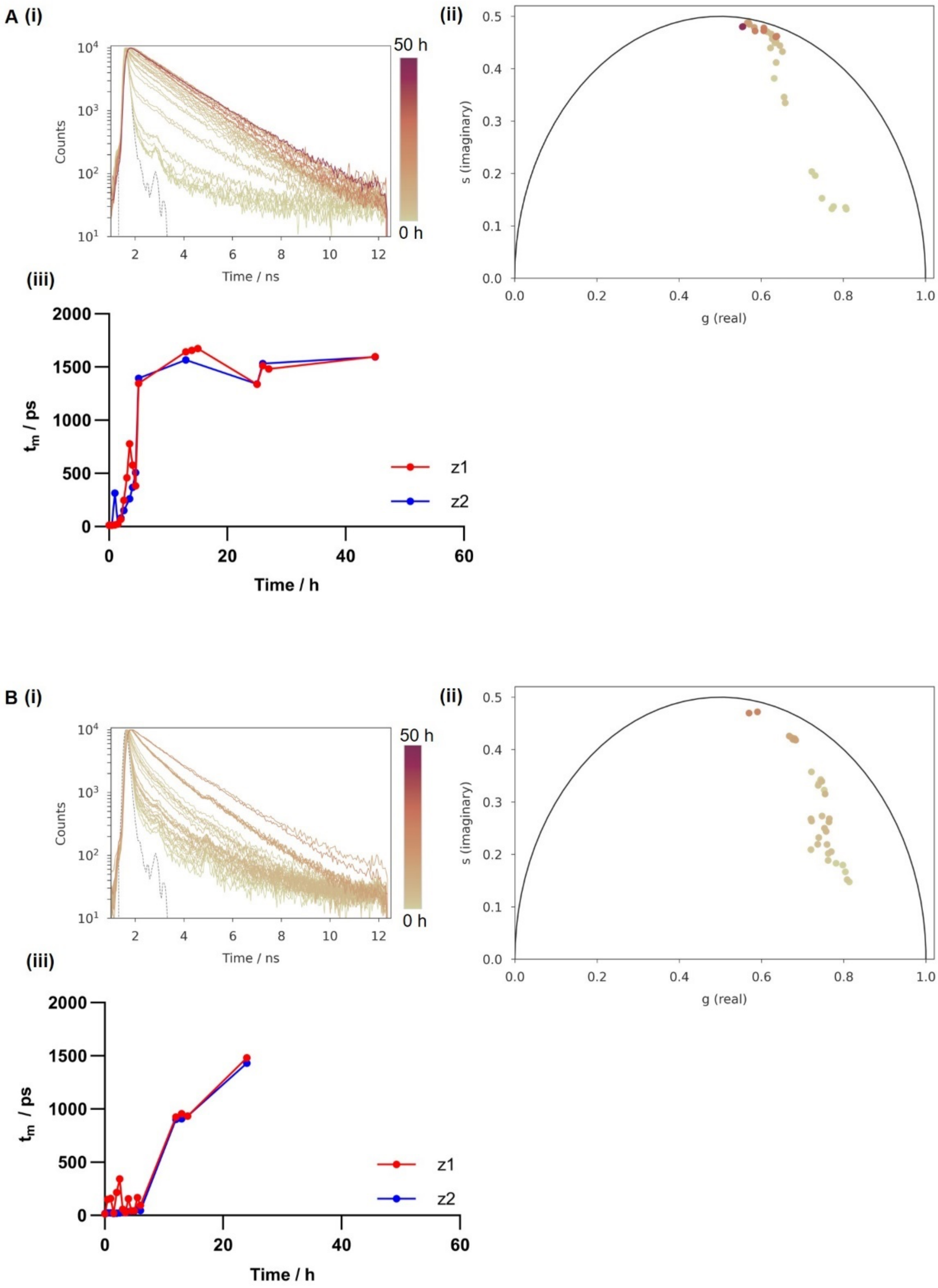
A (i)
Counts
Time / ns
50 h
0 h
(ii)
s (imaginary)
g (real)
(iii)
t_m / ps
Time / h
z1
z2
B (i)
Counts
Time / ns
50 h
0 h
(ii)
s (imaginary)
g (real)
(iii)
t_m / ps
Time / h
z1
z2

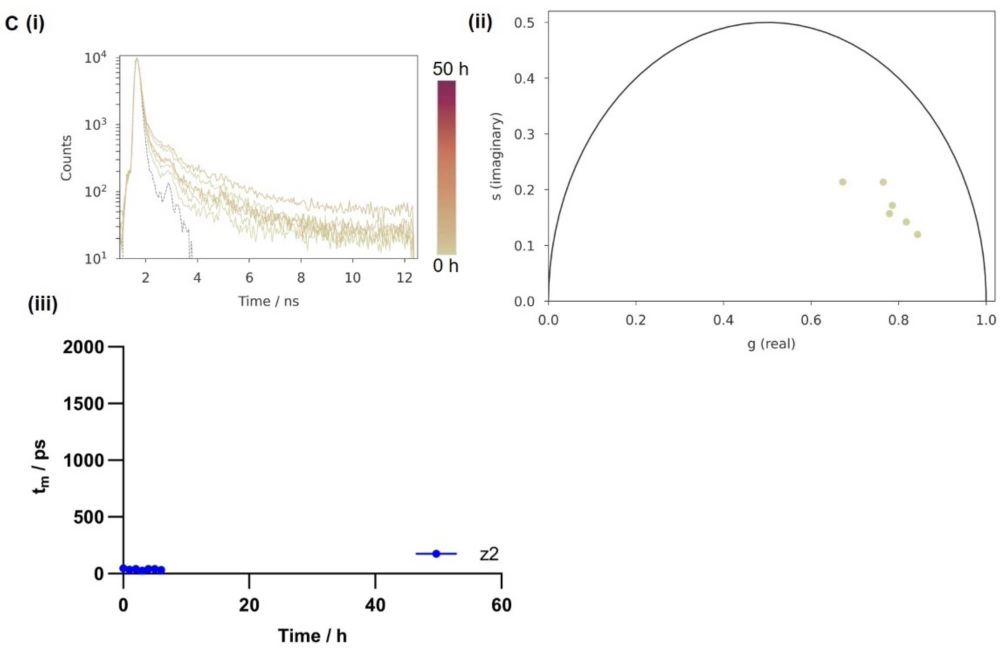
C (i)
(ii)
(iii)
Counts
Time / ns
50 h
0 h
s (imaginary)
g (real)
t_m / ps
Time / h
z2

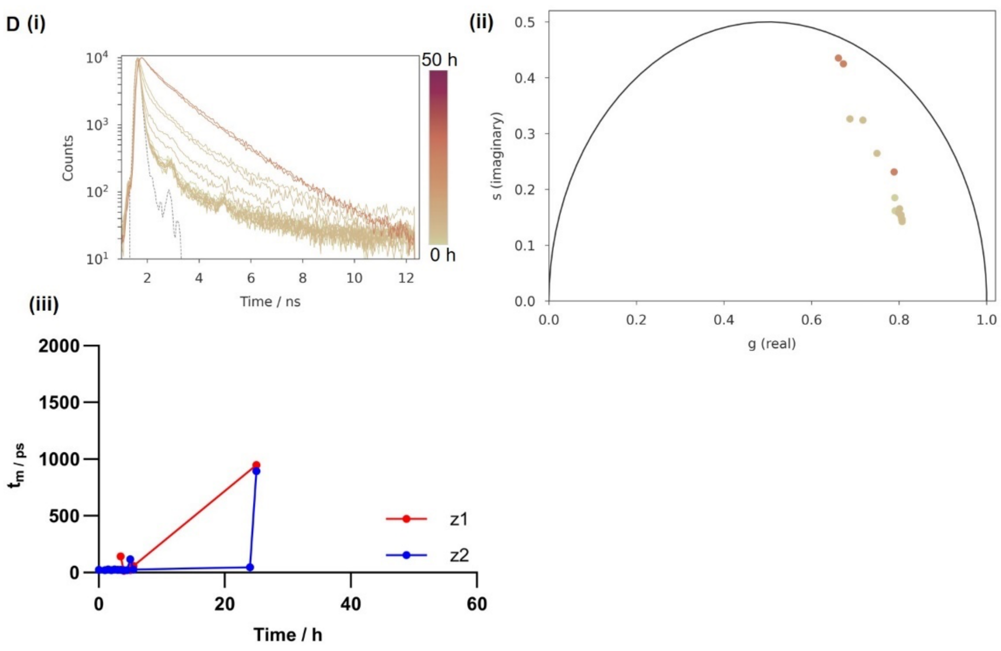
D (i)
(ii)
(iii)
Counts
Time / ns
50 h
0 h
s (imaginary)
g (real)
t_m / ps
Time / h
z1
z2

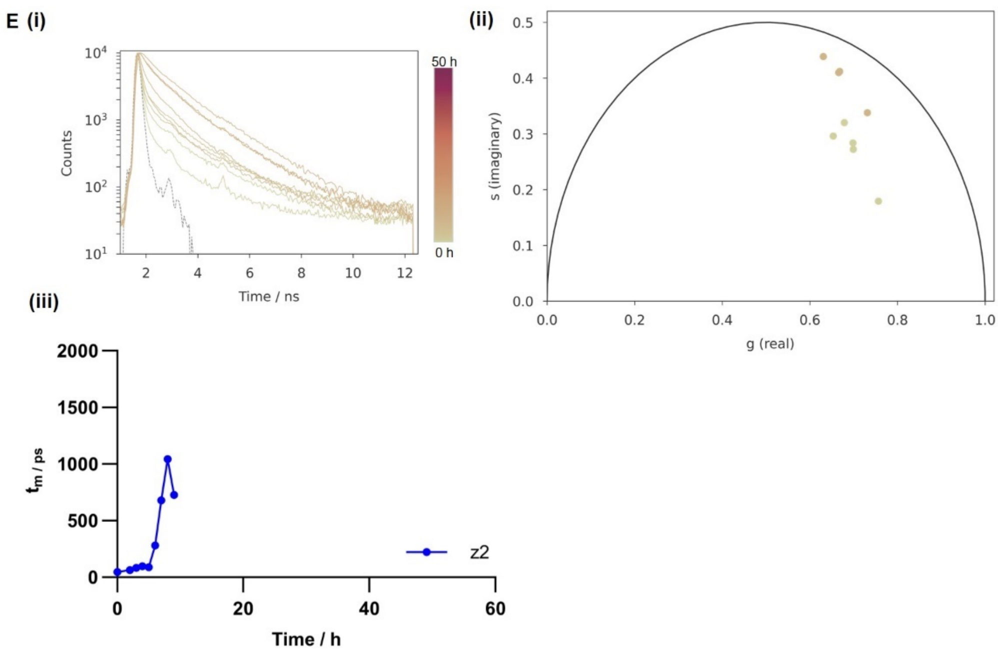

Figure S5. Repeats of ThT time-resolved fluorescence decays and lifetime analysis used to monitor WT αSyn aggregation in PBS (pH 7.4) (A–E). (i) Time-resolved fluorescence decays of ThT (10 µM) in the presence of aggregating WT αSyn (150 µM). (ii) Phasor analysis of ThT decay profile. (iii) Fitted lifetimes (τ_m) of ThT during the aggregation. z-positions were taken at ~100 nm above the well plate surface (z2) and ~1000 nm above this position (z1).

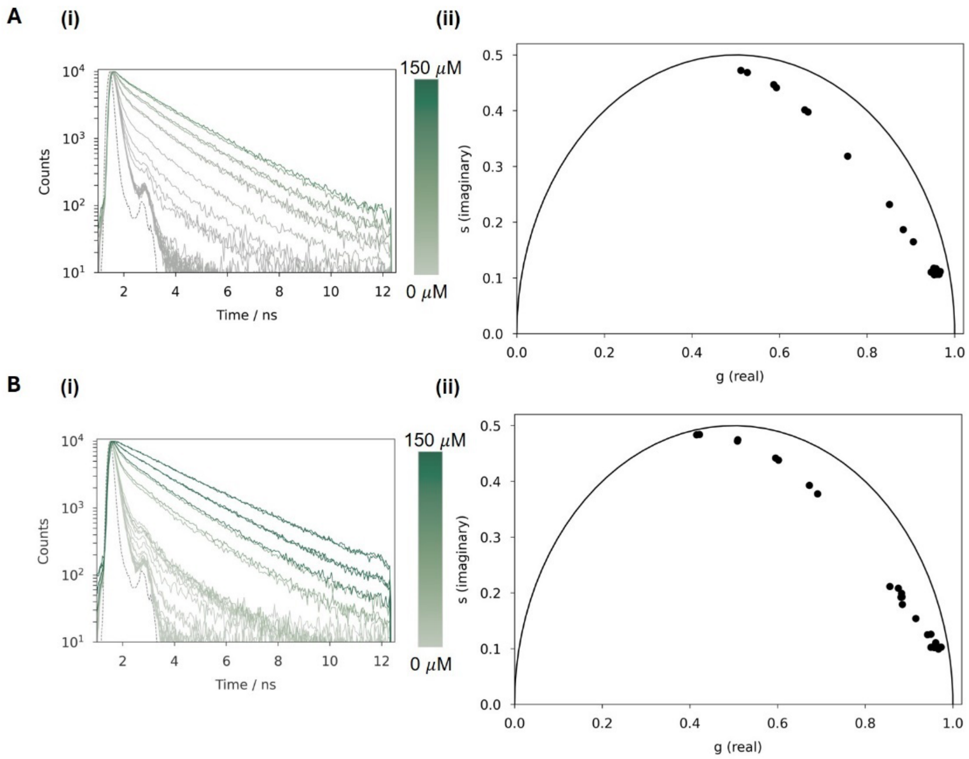

Figure S6. Fluorescence lifetime analysis of $DiSC_2$ in the presence of isolated WT αSyn fibrils in PBS (pH 7.4) (A, B). (i) Time-resolved fluorescence decays of $DiSC_2$ (3 μM) in the presence of increasing fibril concentration (0.001–150 μM) (ii) Phasor analysis of $DiSC_2$ decay profile.

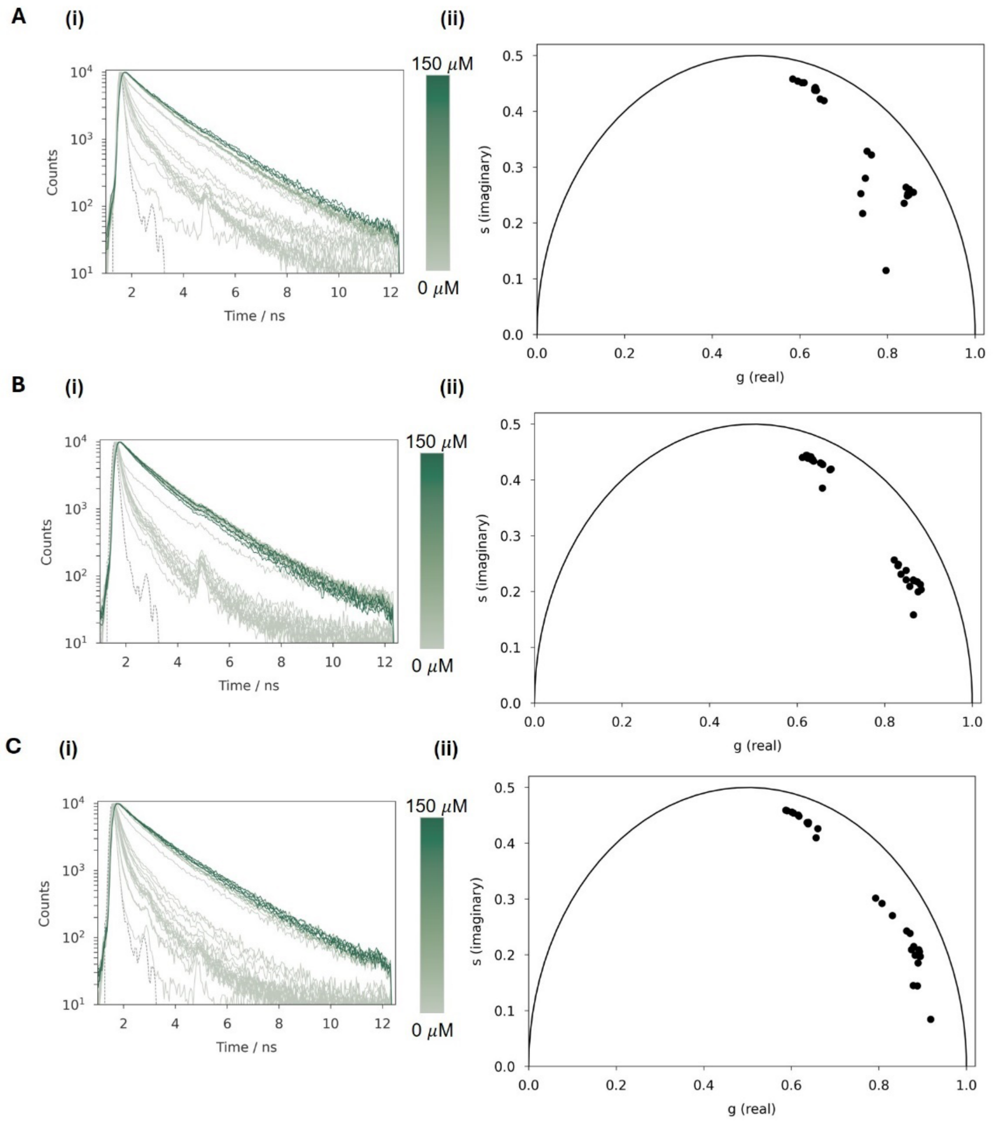

Figure S7. Fluorescence lifetime analysis of ThT in the presence of isolated WT αSyn fibrils in PBS (pH 7.4) (A–C). (i) Time-resolved fluorescence decays of ThT (10 μM) in the presence of increasing fibril concentration (0.001–150 μM) (ii) Phasor analysis of ThT decay profile.

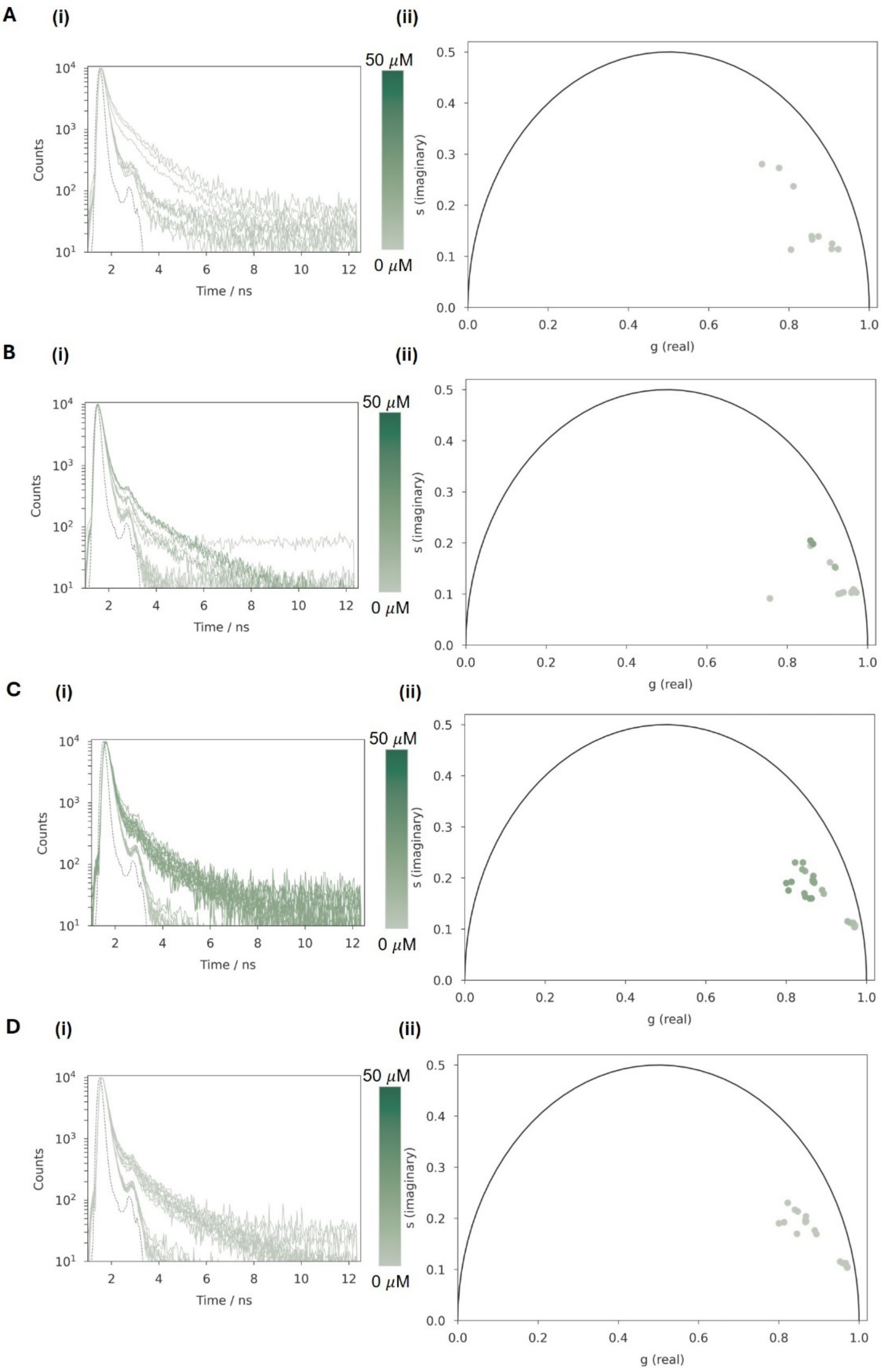

Figure S8. Fluorescence lifetime analysis of $DiSC_2$ in the presence of isolated WT αSyn stabilized oligomers in PBS (pH 7.4) (A–D). (i) Time-resolved fluorescence decays of $DiSC_2$ (3 μM) in the presence of increasing stabilized oligomer concentration (0.001–20 μM) (ii) Phasor analysis of $DiSC_2$ decay profile.

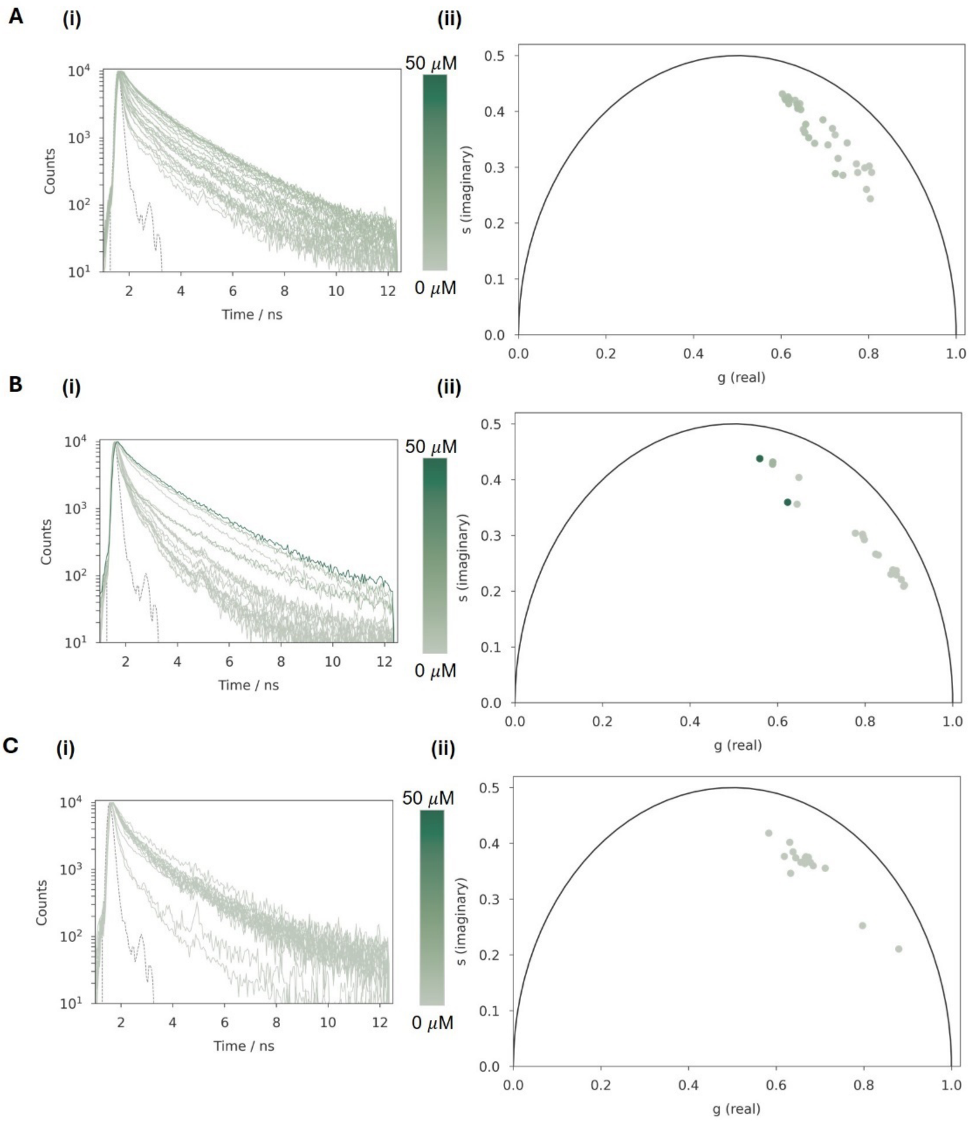

Figure S9. Fluorescence lifetime analysis of ThT in the presence of isolated WT αSyn stabilized oligomers in PBS (pH 7.4) (A–C). (i) Time-resolved fluorescence decays of ThT (10 μM) in the presence of increasing stabilized oligomer concentration (0.001–50 μM) (ii) Phasor analysis of ThT decay profile.

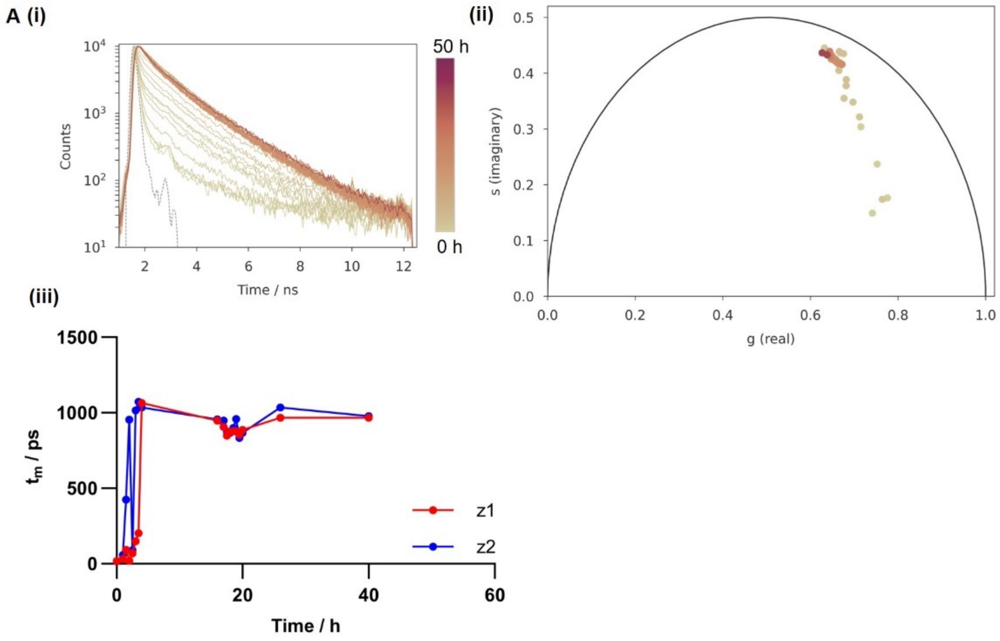
A (i)
(ii)
(iii)
Counts
Time / ns
50 h
0 h
s (imaginary)
g (real)
t_m / ps
Time / h
z1
z2

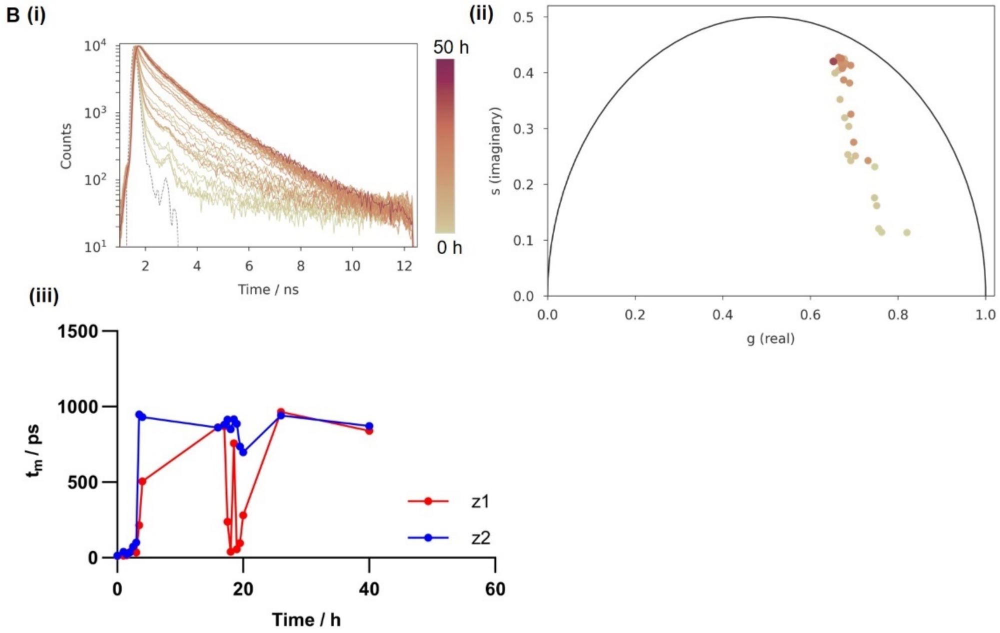
B (i)
(ii)
(iii)
Counts
Time / ns
50 h
0 h
s (imaginary)
g (real)
t_m / ps
Time / h
z1
z2

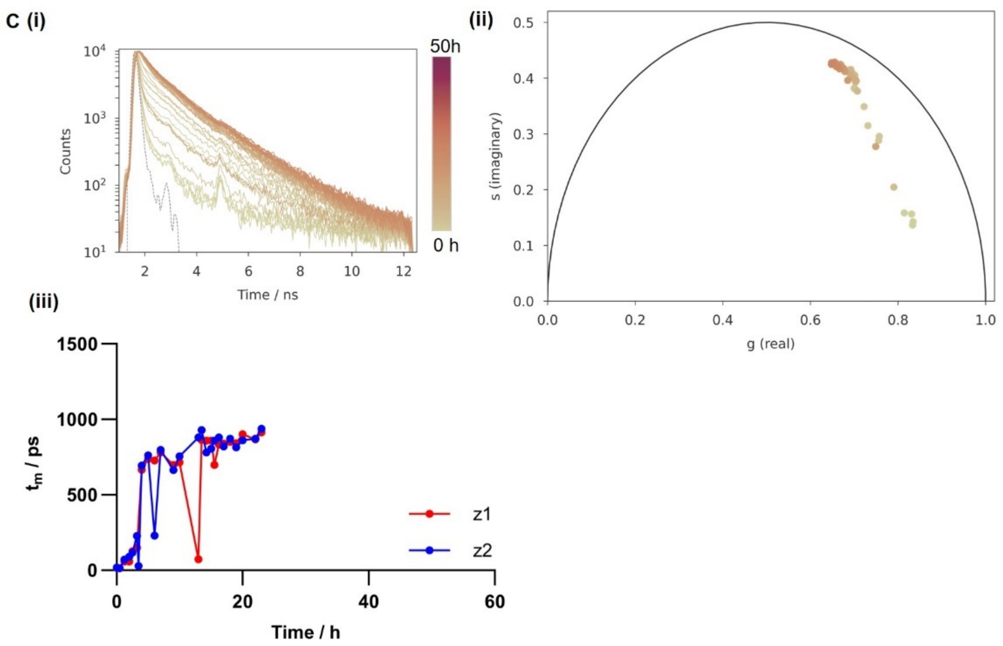

Figure S10. Repeats of ThT time-resolved fluorescence decays and lifetime analysis used to monitor A30P αSyn aggregation in PBS (pH 7.4) (A–C). (i) Time-resolved fluorescence decays of ThT (10 µM) in the presence of aggregating A30P αSyn (150 µM). (ii) Phasor analysis of ThT decay profile. (iii) Fitted lifetimes (τ_m) of ThT during the aggregation. z-positions were taken at ~100 nm above the well plate surface (z2) and ~1000 nm above this position (z1).

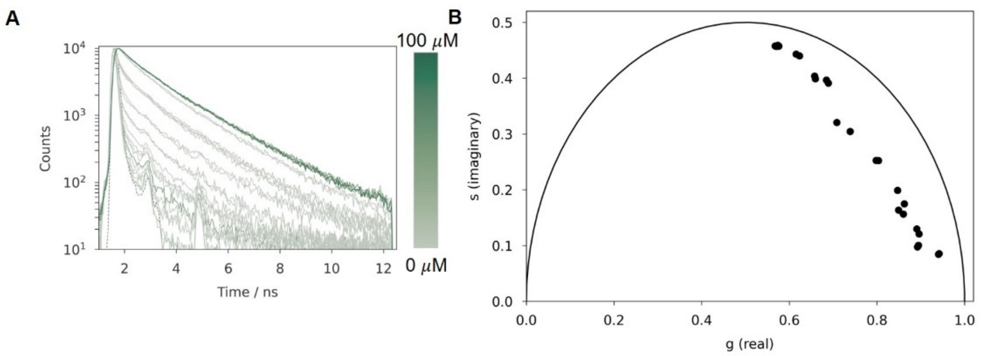

Figure S11. Fluorescence lifetime analysis of ThT in the presence of isolated A30P αSyn fibrils in PBS (pH 7.4). (A) Time-resolved fluorescence decays of ThT (10 µM) in the presence of increasing fibril concentration (0.001–100 µM). (B) Phasor analysis of ThT decay profile.

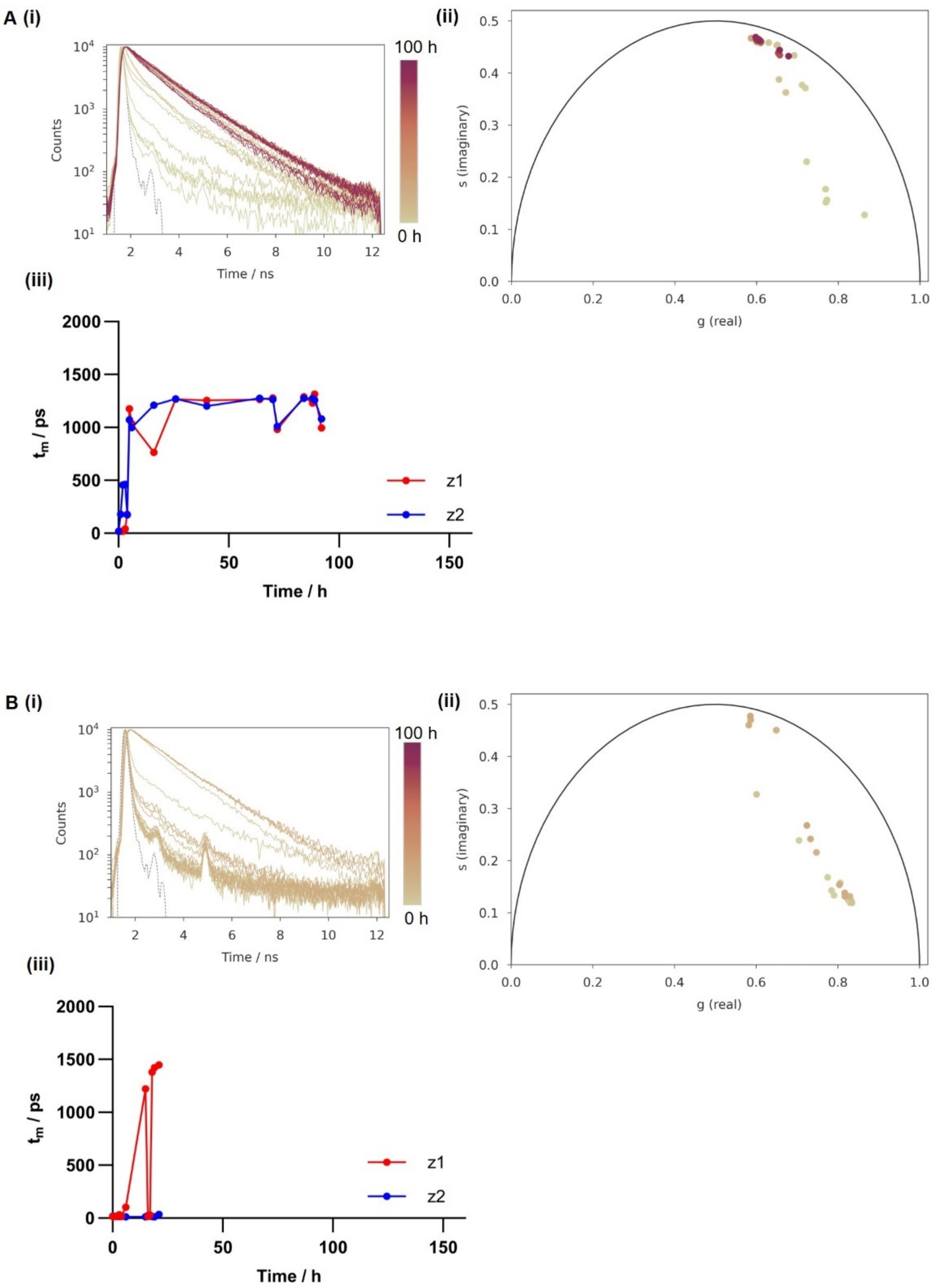
A (i)
(ii)
(iii)
Counts
Time / ns
100 h
0 h
s (imaginary)
g (real)
t_m / ps
Time / h
z1
z2
B (i)
(ii)
(iii)
Counts
Time / ns
100 h
0 h
s (imaginary)
g (real)
t_m / ps
Time / h
z1
z2

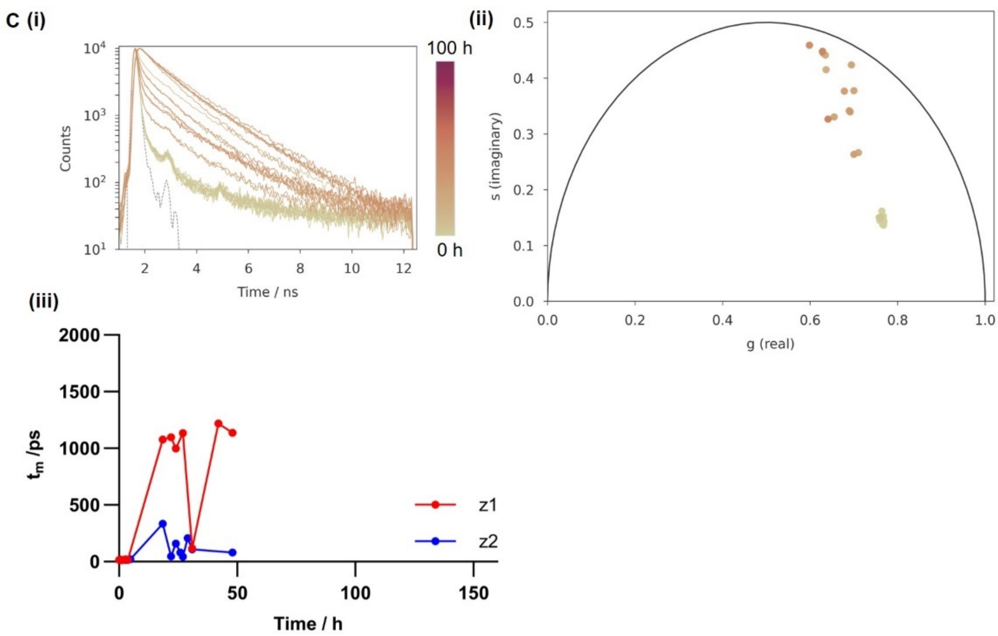

Figure S12. Repeats of ThT time-resolved fluorescence decays and lifetime analysis used to monitor WT αSyn aggregation in 20 mM Tris-HCl with 200 mM NaCl (pH 7.5) (A–C). (i) Time-resolved fluorescence decays of ThT (10 µM) in the presence of aggregating WT αSyn (100 µM). (ii) Phasor analysis of ThT decay profile. (iii) Fitted lifetimes (τ_m) of ThT during the aggregation. z-positions were taken at ~100 nm above the well plate surface (z2) and ~1000 nm above this position (or until the sample surface was reached) (z1).

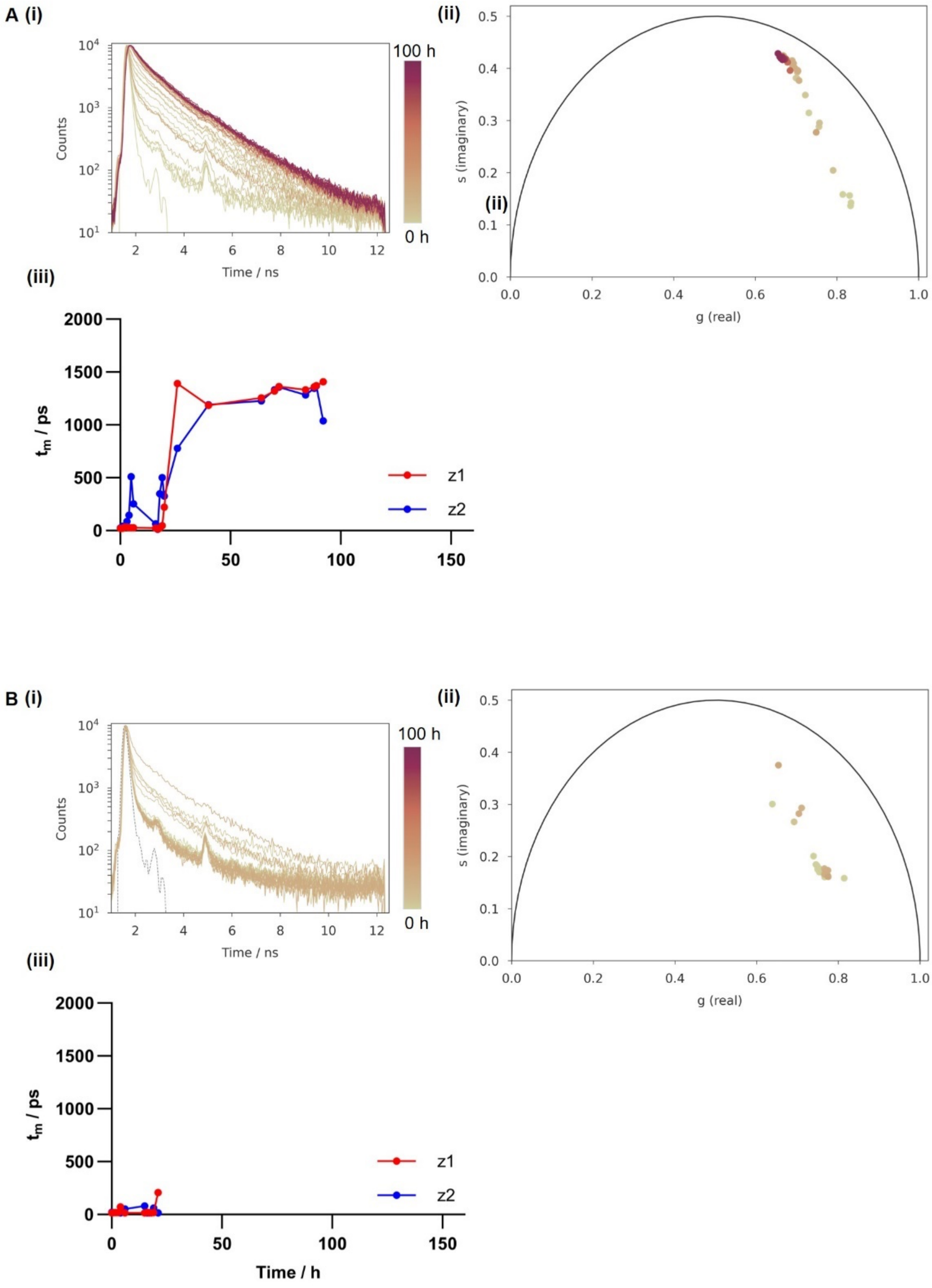

A (i)
(ii)
(iii)
Counts
Time / ns
100 h
0 h
s (imaginary)
g (real)
t_m / ps
z1
z2
B (i)
(ii)
(iii)
Counts
Time / ns
100 h
0 h
s (imaginary)
g (real)
t_m / ps
z1
z2
Time / h

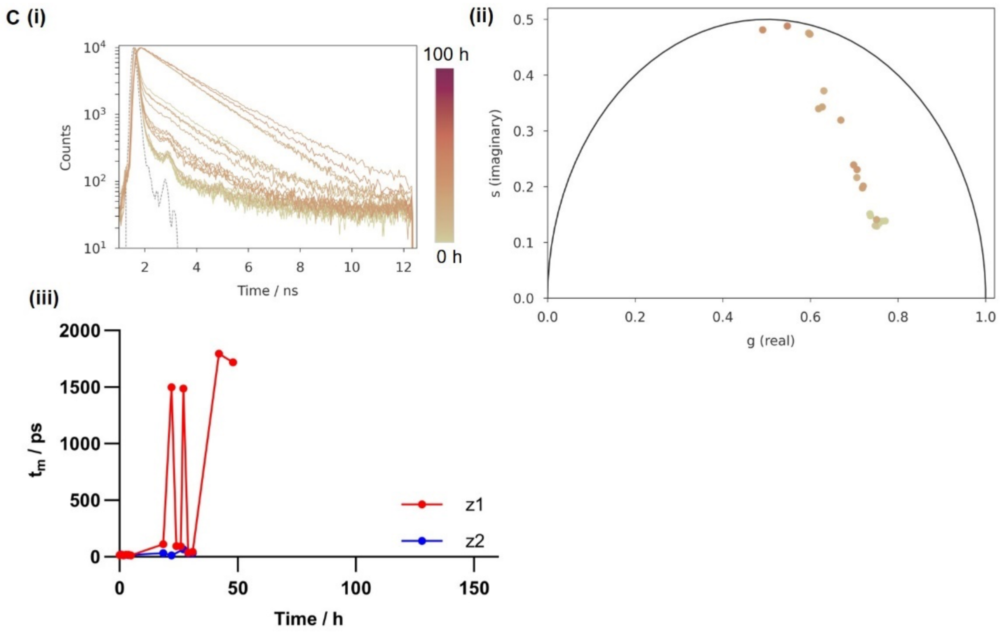

Figure S13. Repeats of ThT time-resolved fluorescence decays and lifetime analysis used to monitor ΔP1 αSyn aggregation in 20 mM Tris-HCl with 200 mM NaCl (pH 7.5) (A–C). (i) Time-resolved fluorescence decays of ThT (10 µM) in the presence of aggregating ΔP1 αSyn (100 µM). (ii) Phasor analysis of ThT decay profile. (iii) Fitted lifetimes (τ_m) of ThT during the aggregation. z-positions were taken at ~100 nm above the well plate surface (z2) and ~1000 nm above this position (or until the sample surface was reached) (z1).

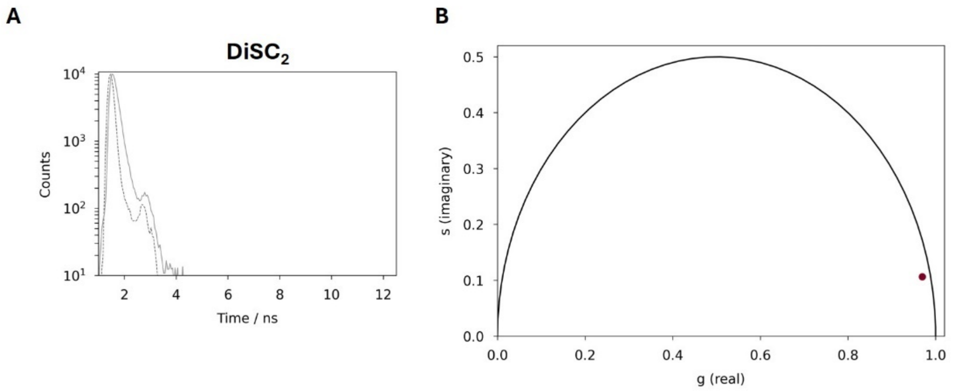

Figure S14. Fluorescence lifetime analysis of free $DiSC_2$ in PBS (pH 7.4). (A) Time-resolved fluorescence decay of free $DiSC_2$ (3 μM). (B) Phasor analysis of $DiSC_2$ decay profile.

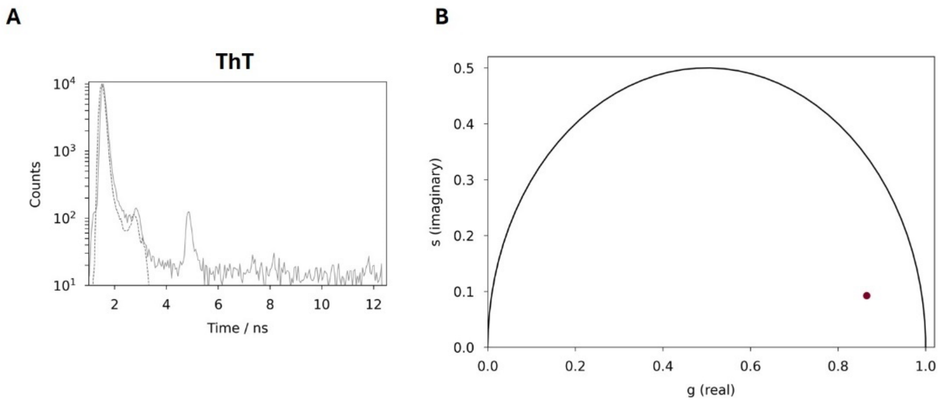

Figure S15. Fluorescence lifetime analysis of free ThT in PBS (pH 7.4). (A) Time-resolved fluorescence decay of free ThT (10 µM). (B) Phasor analysis of ThT decay profile.

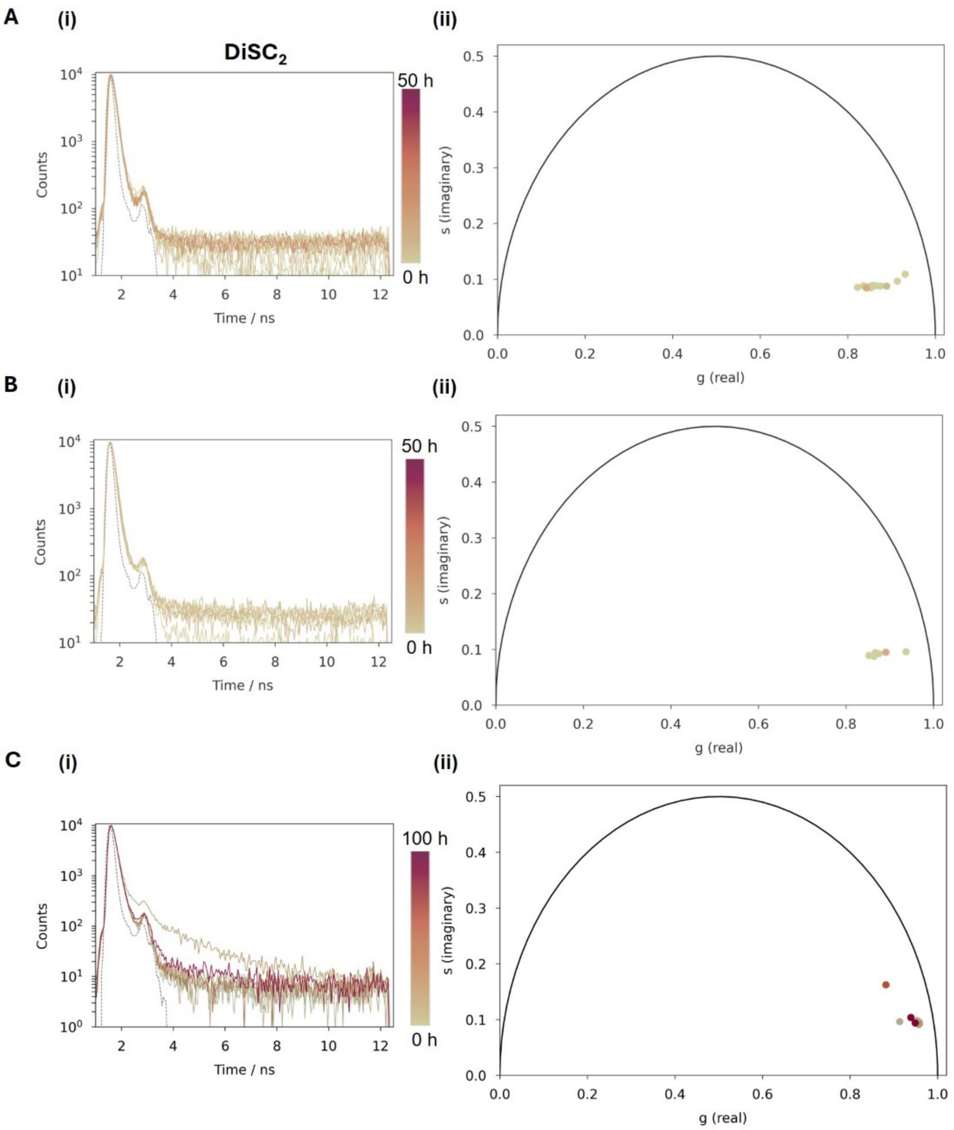

Figure S16. $DiSC_2$ time-resolved fluorescence decays and phasor analysis in PBS (pH 7.4) (A–C). (i) Time-resolved fluorescence decays of $DiSC_2$ (3 μM) in PBS (pH 7.4) over 27 h (A), 24 h (B) and 90 h (C). (ii) Phasor analysis of $DiSC_2$ decay profile.

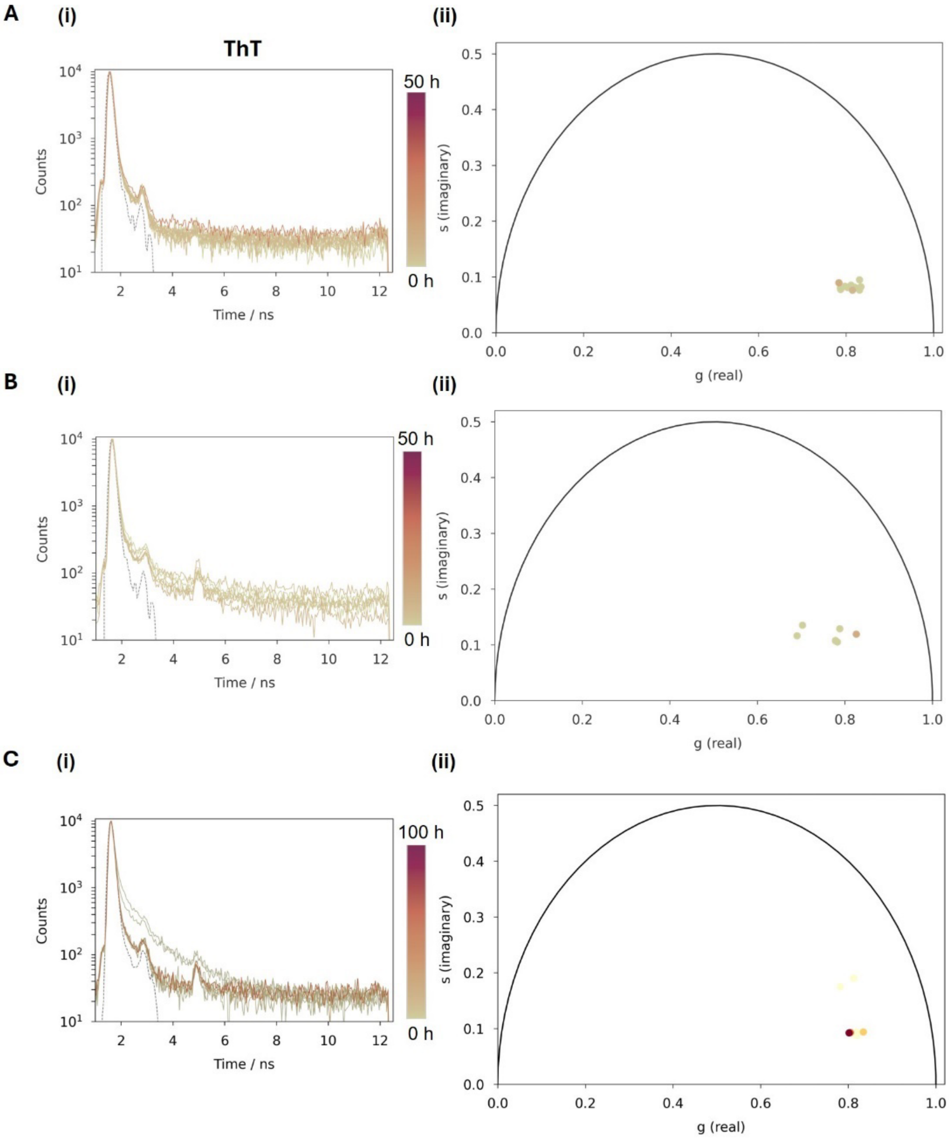

Figure S17. ThT time-resolved fluorescence decays and phasor analysis in PBS (pH 7.4) (A–C). (i) Time-resolved fluorescence decays of ThT (10 μM) in PBS (pH 7.4) over 27 h (A), 24 h (B) and 90 h (C). (ii) Phasor analysis of ThT decay profile.

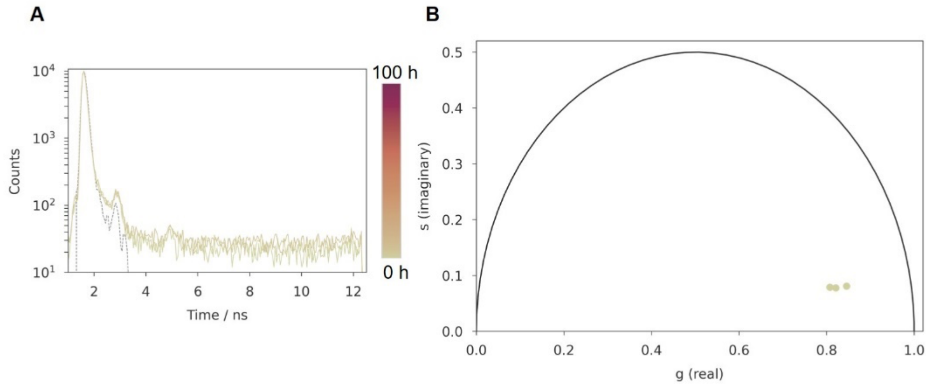

Figure S18. ThT time-resolved fluorescence decays and phasor analysis in 20 mM Tris-HCl with 200 mM NaCl (pH 7.5). (A) Time-resolved fluorescence decays of ThT (10 µM) in 20 mM Tris-HCl with 200 mM NaCl (pH 7.5) over 26 h. (B) Phasor analysis of ThT decay profile.

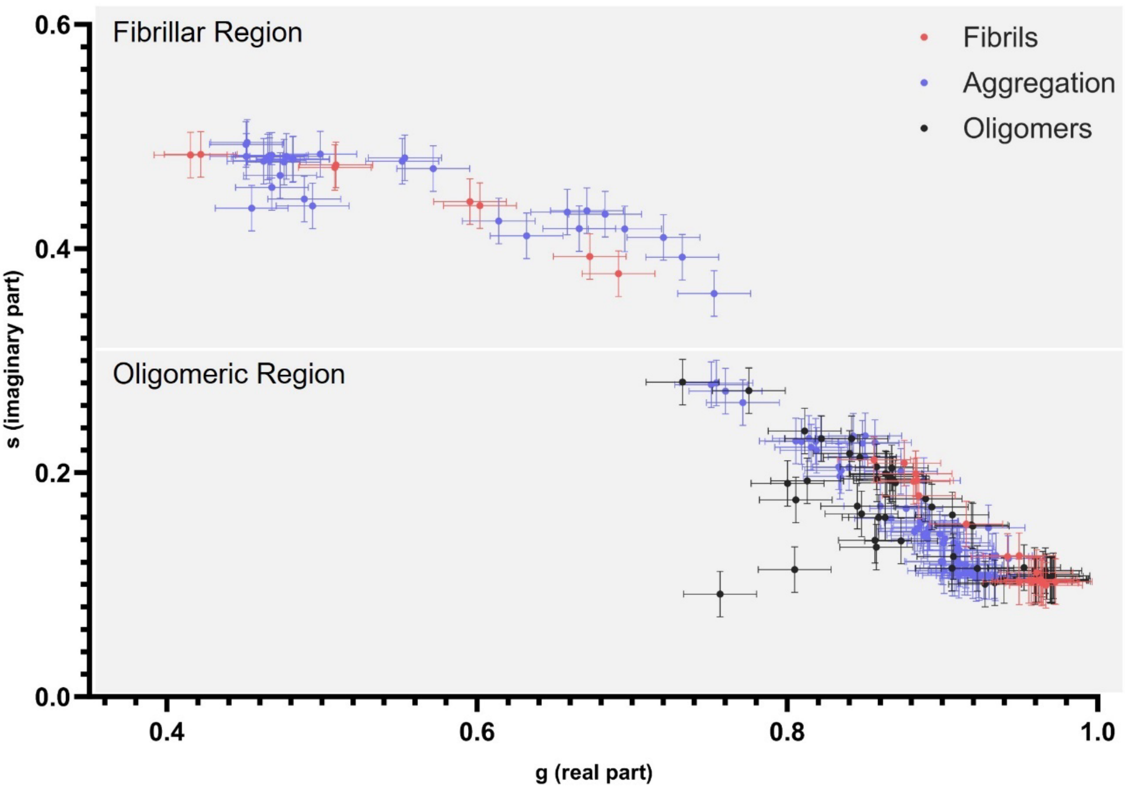

Figure S19. Classification of the separate regions of the $DiSC_2$ phasor plot. Error bars represent the standard deviation (2σ) of technical repeats of FLIM fibril and oligomer measurements. Two points are considered overlapping if there is overlap in two error bar directions. Regions are assigned if there is at least a 60 % overlap between points. In this case ~70% of aggregation points in the fibrillar region overlap with the fibrillar points. In the oligomeric region, ~60% of the aggregation points deviate from the fibrillar region and all the stabilized oligomers reside in this region, with all the aggregation data points overlapping with the stabilized oligomers.

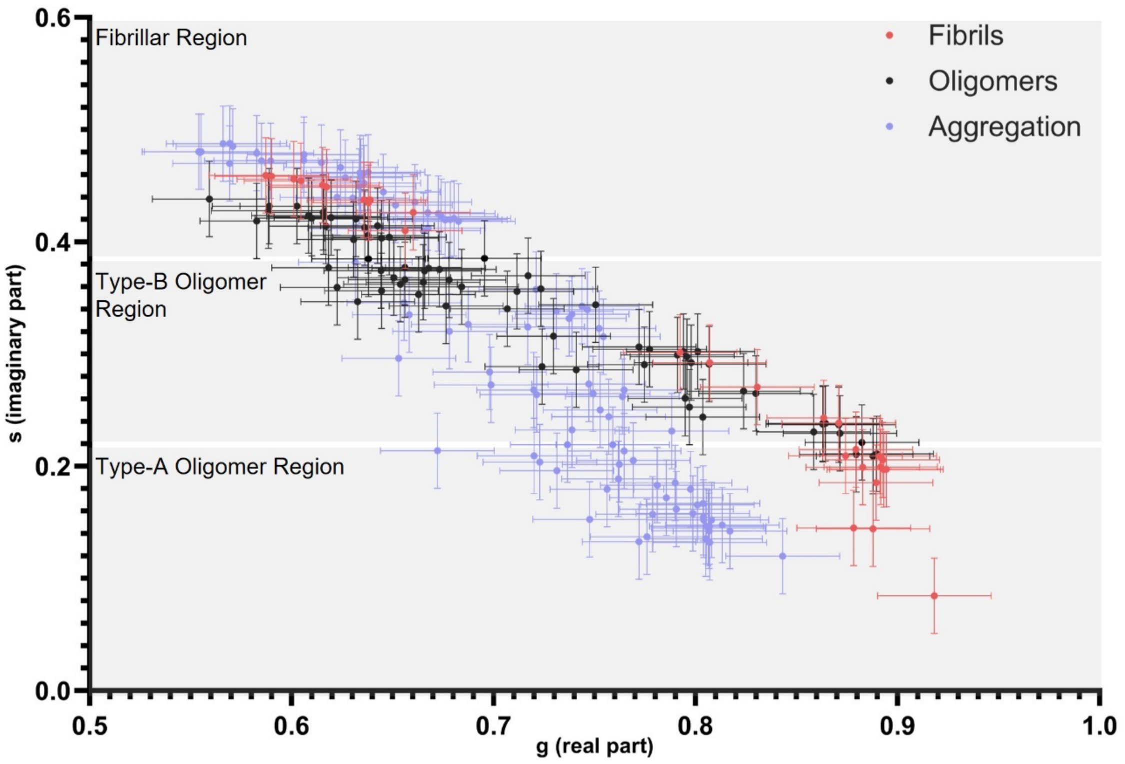

Figure S20. Classification of the separate regions of the ThT phasor plot. Error bars represent the standard deviation (2σ) of technical repeats of FLIM fibril and oligomer measurements. Two points are considered overlapping if there is overlap in two error bar directions. Regions are assigned if there is at least a 60% overlap between points. In this case, ~94% of aggregation points in the fibrillar region overlap with the fibrillar points. In the Type-B oligomer region ~69% aggregation points overlap with the stabilized oligomer points and in the Type-A region there is less than 4 % overlap of the aggregation data with either fibrillar or stabilized oligomer points.

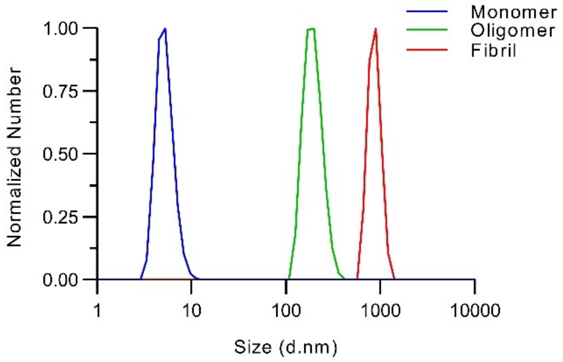

Figure S21. DLS analysis of monomeric (blue), oligomeric (green) and fibrillar WT αSyn (red).

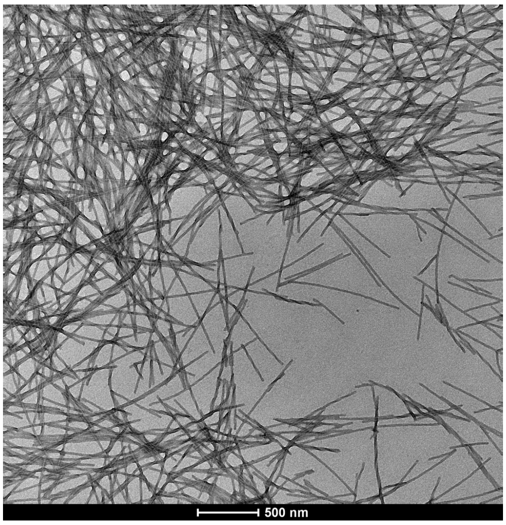

Figure S22. Representative TEM image of fully formed WT αSyn fibrils (150 μM).

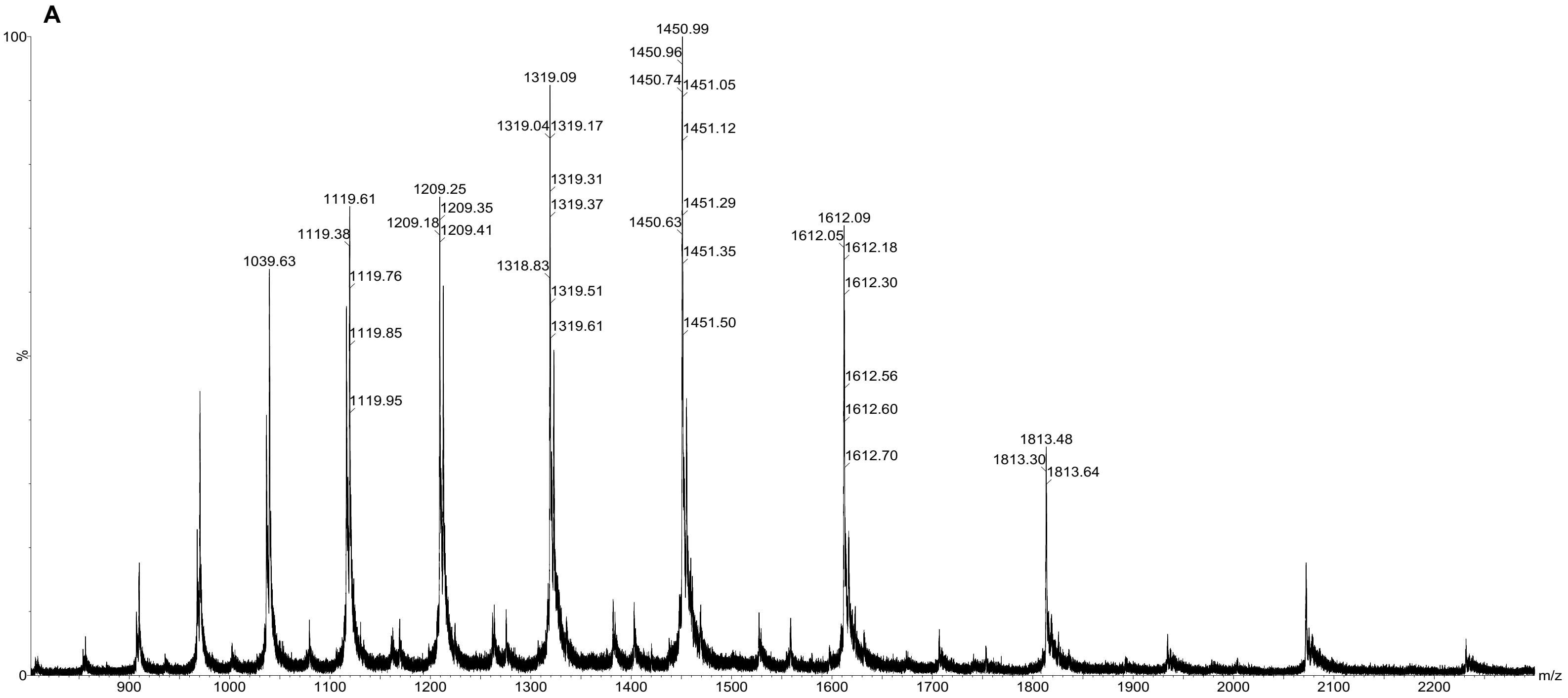

A
100
%
0
1450.99
1450.96
1450.74
1451.05
1451.12
1451.29
1450.63
1451.35
1451.50
1319.09
1319.04
1319.17
1319.31
1319.37
1318.83
1319.51
1319.61
1209.25
1209.35
1209.18
1209.41
1119.61
1119.38
1119.76
1119.85
1119.95
1039.63
1612.09
1612.05
1612.18
1612.30
1612.56
1612.60
1612.70
1813.48
1813.30
1813.64
900
1000
1100
1200
1300
1400
1500
1600
1700
1800
1900
2000
2100
2200
m/z

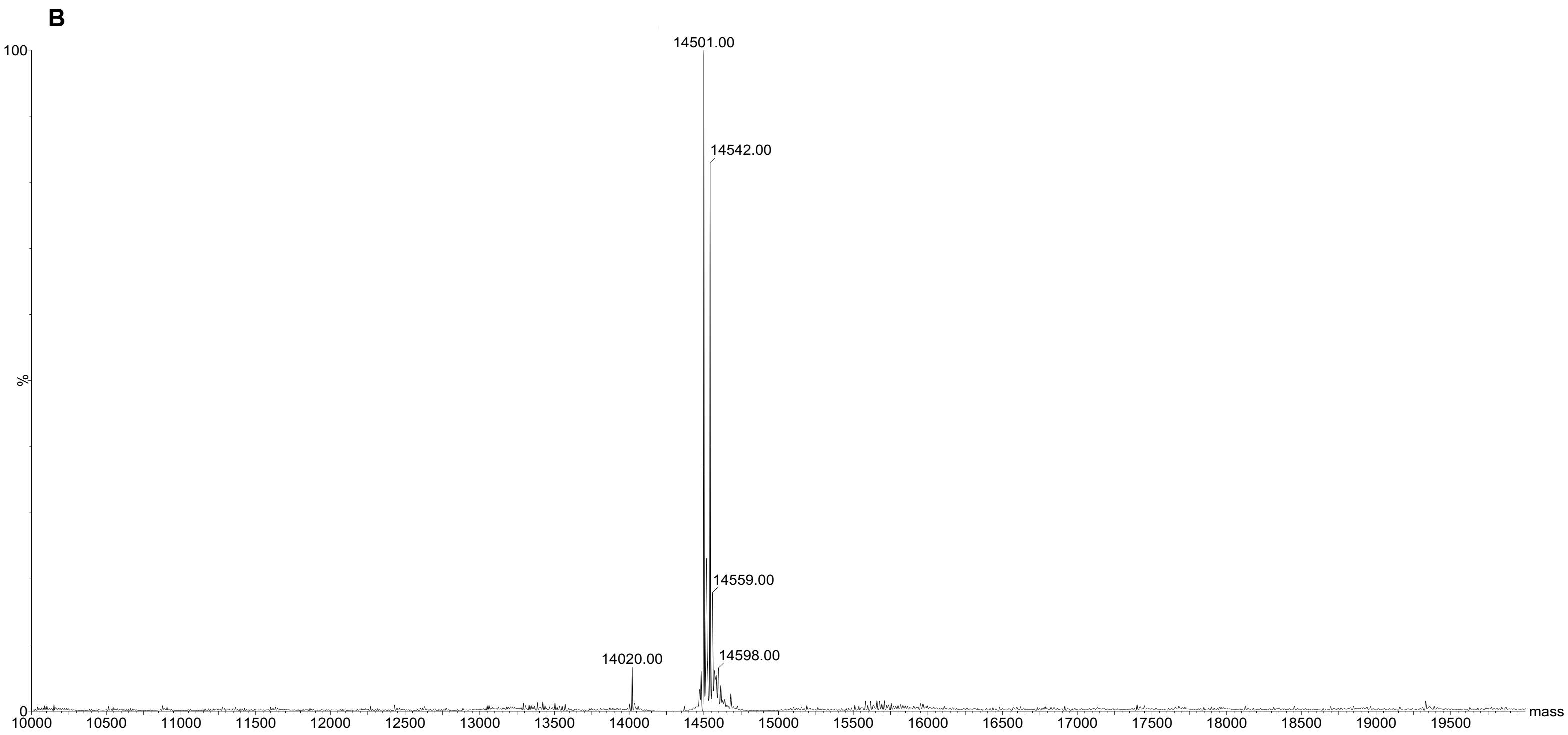

Figure S23. ESI-TOF MS spectrum (A) and deconvolution data (B) of pure WT αSyn in water. The spectrum shows peaks at +41, +82, +99 and +138 relative to the expected molecular weight. These peaks can likely be attributed to acetonitrile adducts (+ 41) formed during mass spectrometry, in various combinations with oxidized methionine (+16), also potentially due to the mass spectrometry analysis.

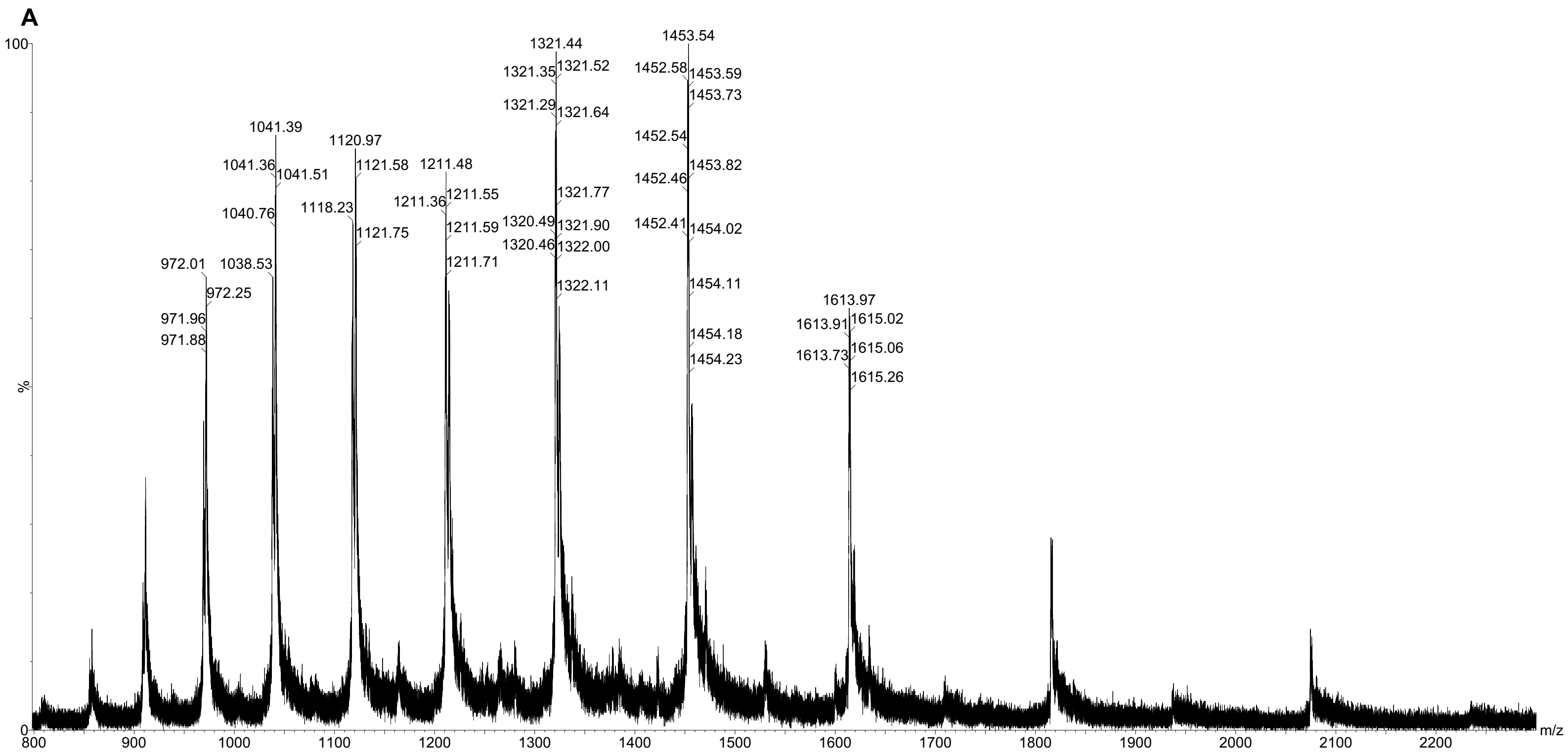
A
100
%
0
1041.39
1120.97
1211.48
1321.44
1453.54
1613.97
972.01
m/z
800
900
1000
1100
1200
1300
1400
1500
1600
1700
1800
1900
2000
2100
2200

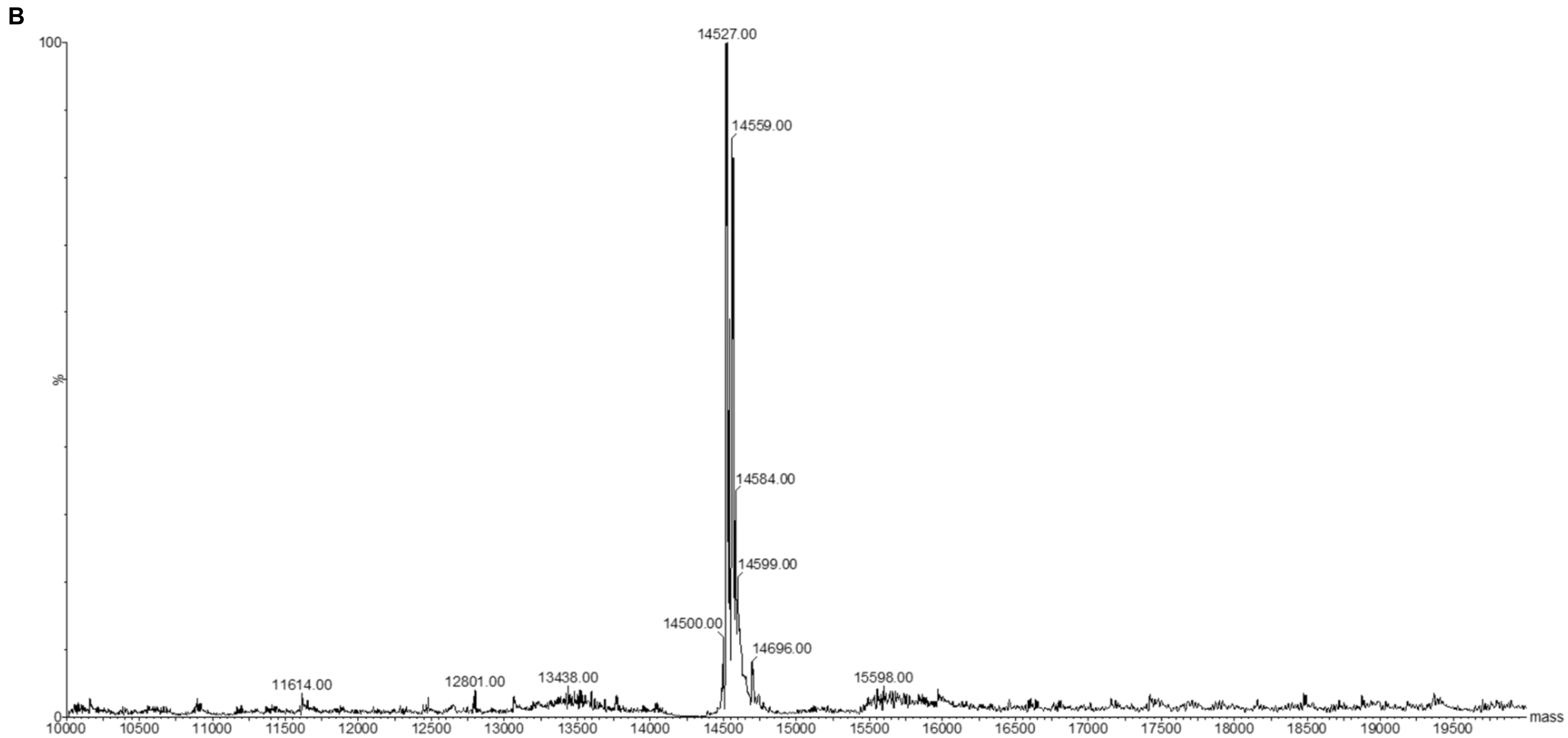

Figure S24. ESI-TOF MS spectrum (A) and deconvolution data (B) analysis of pure A30P αSyn in water. The spectrum shows peaks at +41, +73, +98 and +113 relative to the expected molecular weight. These peaks can likely be attributed to acetonitrile adducts (+ 41) formed during mass spectrometry, in various combinations with oxidized methionine (+16), also potentially due to the mass spectrometry analysis.

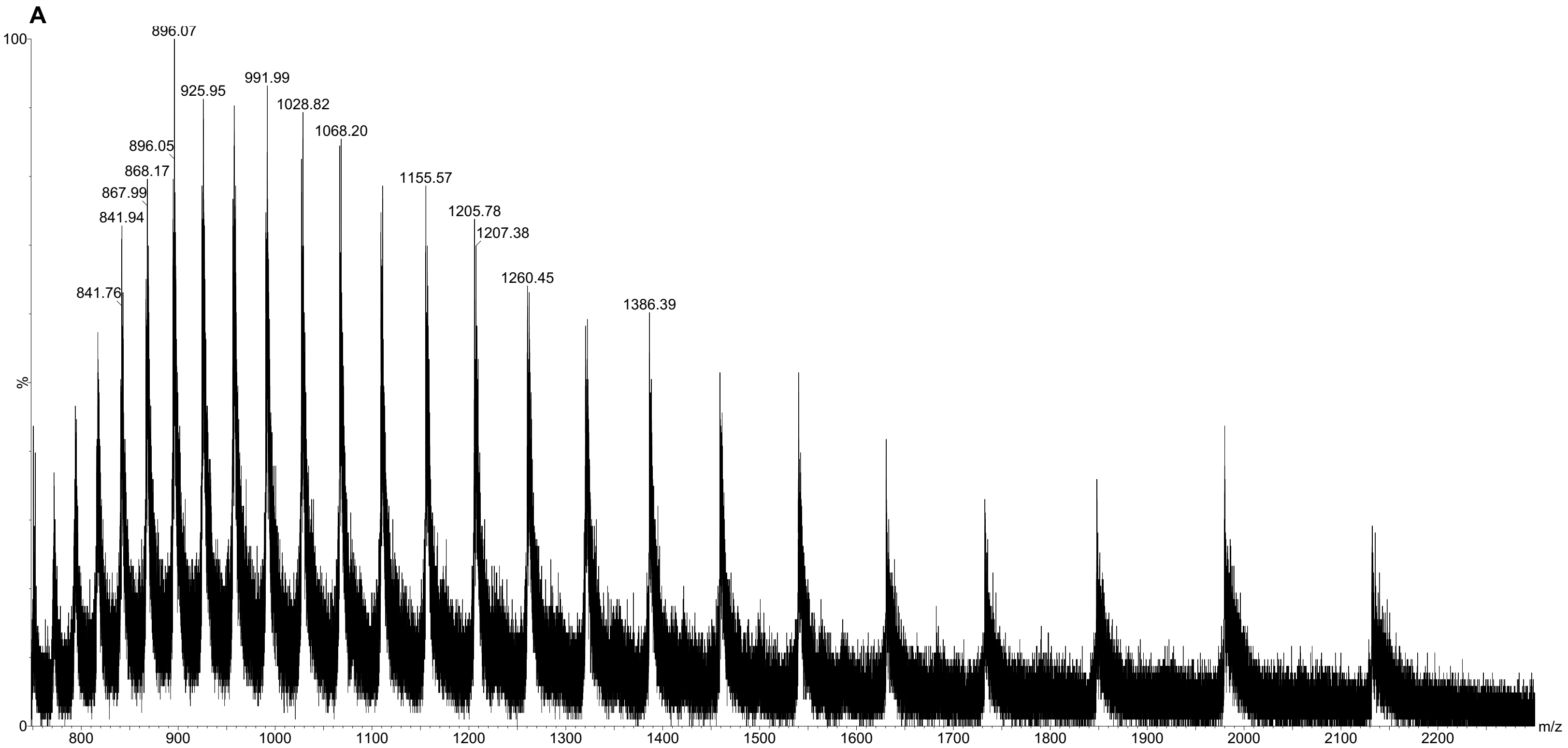
A
100
896.07
925.95
991.99
1028.82
1068.20
896.05
868.17
867.99
841.94
1155.57
1205.78
1207.38
841.76
1260.45
1386.39
%
0
800
900
1000
1100
1200
1300
1400
1500
1600
1700
1800
1900
2000
2100
2200
m/z

B

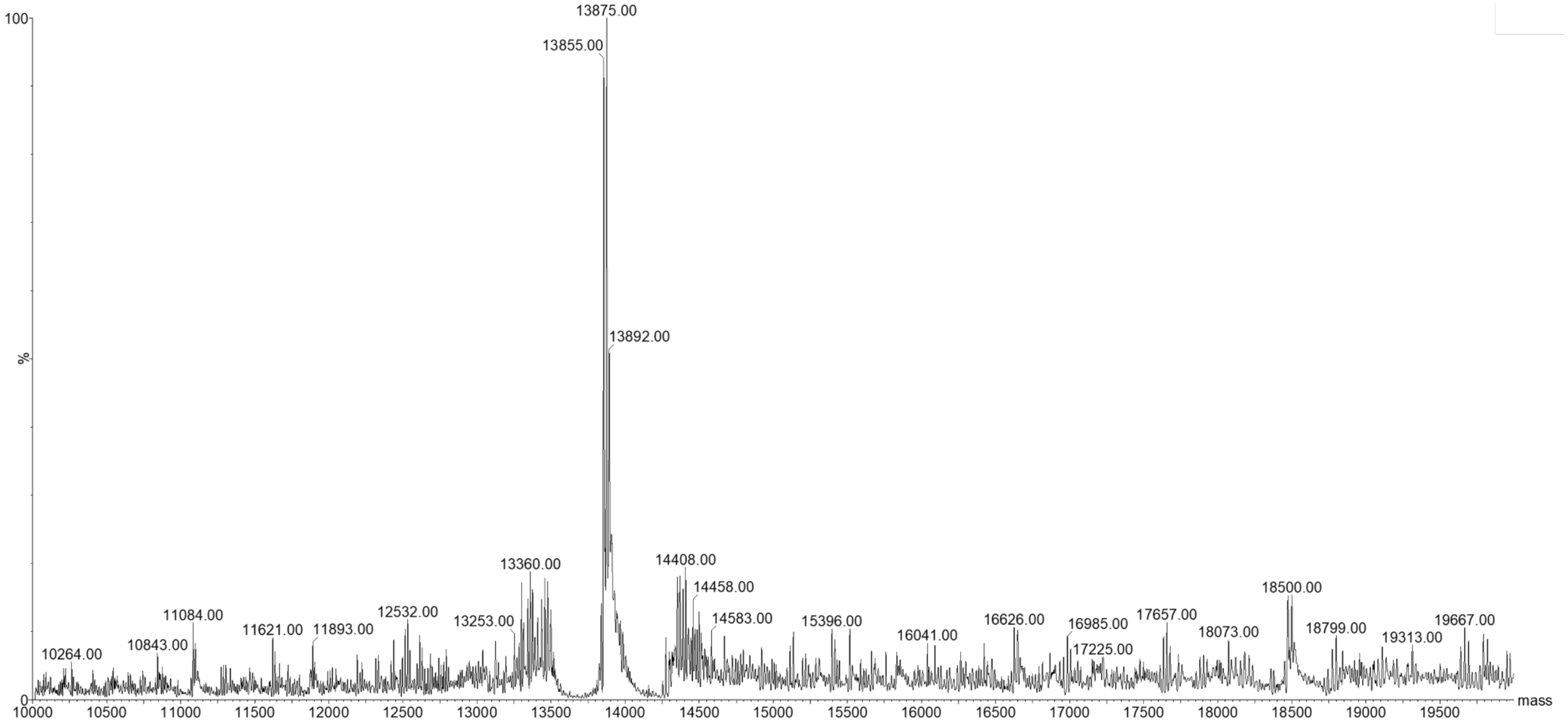

Figure S25. ESI-TOF MS spectrum (A) and deconvolution data (B) of pure ΔP1 αSyn in water. The spectrum shows peaks at +71, +91 and +108 relative to the expected molecular weight. These peaks can likely be attributed to acetonitrile adducts (+ 41) formed during mass spectrometry, in various combinations with oxidized methionine (+16), also potentially due to the mass spectrometry analysis.

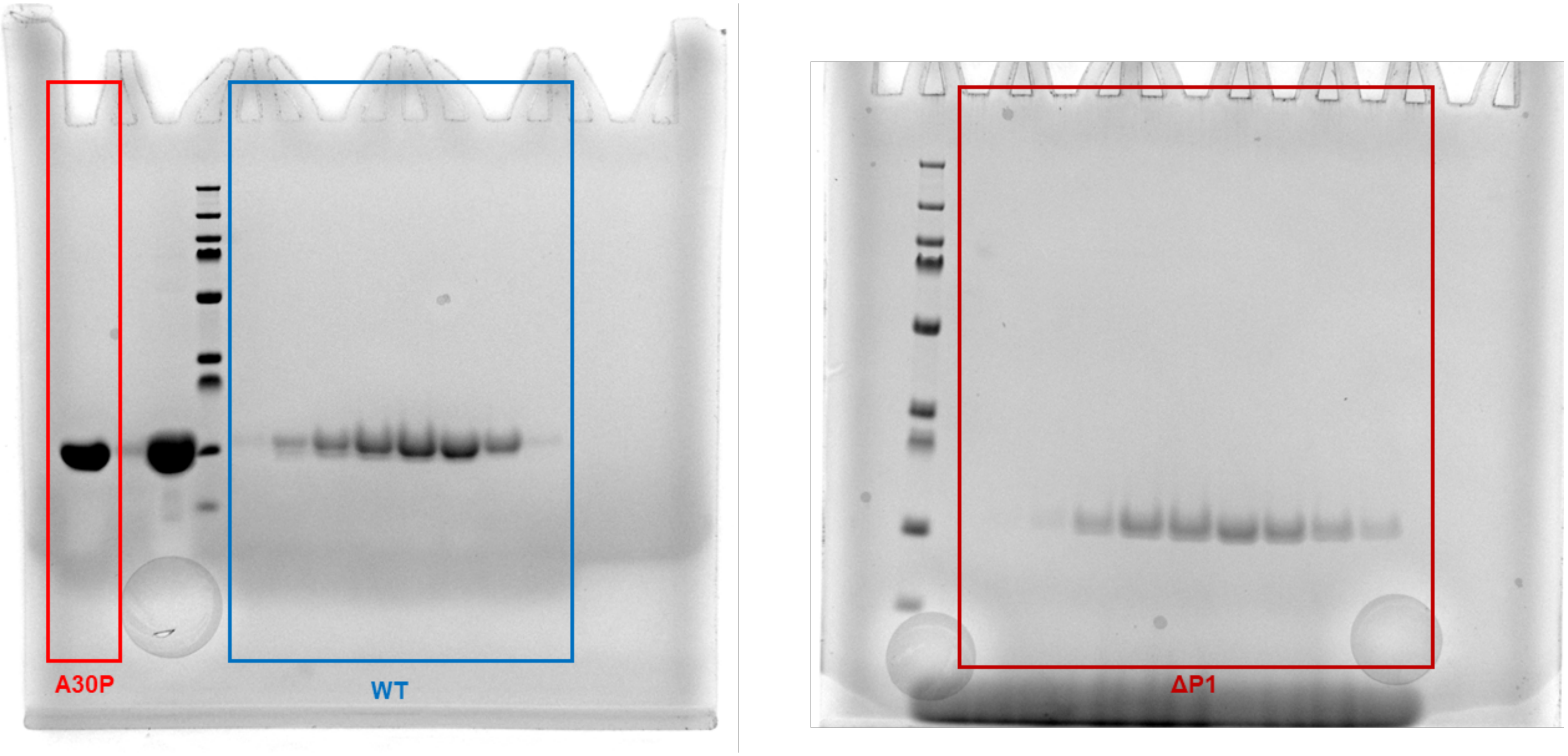

Figure S26. SDS-PAGE of purified A30P, WT and ΔP1 αSyn after SEC. A30P αSyn fractions from SEC have been combined. WT and ΔP1 αSyn fractions from SEC have been imaged before combining.
